# Glycolysis-enhancing α_1_-adrenergic antagonists have therapeutic potential in Alzheimer’s disease

**DOI:** 10.1186/s13195-026-02135-z

**Published:** 2026-07-09

**Authors:** Qiang Zhang, Jordan L Schultz, Jacob E Simmering, Braedon Q Kirkpatrick, Matthew A Weber, Sydney Skuodas, Tara Hicks, Grace Pierce, Margaret Laughlin, Benu George, Aimee X Bertolli, Travis Larson, Ramasamy Thangavel, Mayu Oya, David K Meyerholz, Georgina Aldridge, Jan Fassler, Nandakumar S. Narayanan

**Affiliations:** 1 Department of Neurology, Roy J. and Lucille A. Carver College of Medicine, University of Iowa, 169 Newton Road, Iowa City, IA 52242 USA; 2 Department of Psychiatry, Roy J. and Lucille A. Carver College of Medicine, University of Iowa, Iowa City, IA 52242 USA; 3 Department of Internal Medicine, Roy J. and Lucille A. Carver College of Medicine, University of Iowa, Iowa City, IA 52242 USA; 4 Department of Biology, University of Iowa, Iowa City, IA 52242 USA; 5 Duke Medical Scientist Training Program, Department of Neurobiology, Duke University, Durham, NC 27710 USA; 6 Johns Hopkins University, Baltimore, MD 21205 USA; 7 Department of Pathology, University of Iowa, Iowa City, IA 52242 USA

## Abstract

**Background:**

Terazosin (TZ) is widely prescribed for hypertension and benign prostatic hyperplasia. Recent studies suggest that TZ enhances glycolysis and may protect against neurodegenerative diseases.

**Methods:**

We tested the hypothesis that TZ is neuroprotective in Alzheimer’s disease (AD) in a yeast model, a mouse model, and two human datasets.

**Results:**

We report four main results. First, TZ increased ATP levels in a *Saccharomyces cerevisiae* mutant with impaired metabolism, and reduced Amyloid-beta42 (Aβ42) aggregation. Second, in 5xFAD mice, TZ attenuated amyloid pathology and rescued cognitive impairments in spatial memory and interval timing. Third, in the Alzheimer’s Disease Neuroimaging Initiative (ADNI) database, AD patients taking glycolysis-enhancing drugs had a slower progression of both cognitive dysfunction and metabolic neuroimaging biomarkers (18 F-fluorodeoxyglucose positron emission tomography (FDG-PET)). Finally, in the Merative Marketscan dataset, patients taking glycolysis-enhancing drugs had lower risks of developing AD.

**Conclusion:**

These data provide preliminary evidence that glycolysis-enhancing drugs have therapeutic potential in AD.

**Supplementary Information:**

The online version contains supplementary material available at https://doi.org/10.1186/s13195-026-02135-z.

## Background

Alzheimer’s disease (AD) is the leading cause of dementia and affects over 6 million Americans [1]. The pathophysiology of AD involves complex mechanisms, including amyloid-beta (Aβ) aggregation, tau pathology, and neuronal loss, which cause synaptic dysfunction and cognitive impairment. Despite recent advances [2, 3], there is an ongoing need for better therapies for AD.

A factor that may contribute to AD is impaired energy metabolism [4–6]. Four lines of evidence support this link: (1) Aging, a primary risk factor for AD, is associated with impaired cerebral glucose metabolism, reduced mitochondrial biogenesis, and decreased adenosine 5′-triphosphate (ATP) levels [7, 8]. (2) Diseases of metabolism such as diabetes and obesity increase the risk of AD [4, 6]. (3) Interventions that improve metabolism, such as caloric restriction and exercise, can improve neuropathology and behavior in mouse models of AD and cognitive function in humans with AD [9–11]. (4) AD patients can have changes in ^18^F-fluorodeoxyglucose positron emission tomography (FDG-PET), as glycolysis is reduced in brain areas with amyloid deposition and tau accumulation [12, 13]. This metabolic vulnerability has motivated a broader “type 3 diabetes” framework in which brain insulin resistance, impaired glucose utilization, and disrupted energy sensing through AMPK and mTOR contribute to amyloid accumulation, tau pathology, and synaptic dysfunction [14–16].

Therapeutic strategies targeting metabolism — most prominently GLP-1 receptor agonists — have been evaluated in recent randomized trials with mixed but informative results: the phase 2b ELAD trial of liraglutide showed a ~ 50% reduction in temporal-lobe atrophy and slowed cognitive decline despite missing its primary cerebral glucose metabolism endpoint [17], whereas the EVOKE trials of oral semaglutide did not slow clinical progression despite favorable biomarker movement [18, 19]. These results collectively support the principle that metabolic engagement in AD is achievable but suggest that GLP-1 modulation alone may be insufficient, motivating exploration of more directly bioenergetic strategies. Pharmacologic enhancement of glycolysis represents an attractive but under-explored strategy. Terazosin (TZ), an FDA-approved α1-adrenergic antagonist, was discovered to bind and activate phosphoglycerate kinase 1 (PGK1) — the first ATP-generating enzyme in glycolysis — and increase cellular ATP, an effect subsequently shown to slow neurodegeneration in Parkinson’s disease models and to be associated with reduced PD incidence and slower progression in human cohorts [20–24]. TZ was also shown to mitigate neurodegeneration in the APPsw/PS1 transgenic mouse model of AD [25].

We hypothesized that the glycolysis-enhancing activity of TZ has neuroprotective potential in AD. We tested this hypothesis in yeast models, in a mouse model of AD, in humans from the Alzheimer’s Disease Neuroimaging Initiative (ADNI) cohort, and in a large database of health insurance claims. These findings contribute to the growing body of evidence focused on metabolism in neurodegenerative disease and highlight the potential of glycolysis-enhancing drugs as a treatment for patients with AD.

## Methods

### Yeast

#### Strains

We studied metabolism in wild type (WT) strains and strains of yeast lacking the SNF1 gene, which encodes the yeast homolog of the catalytic subunit of the AMP-activated protein kinase (AMPK) complex, a central regulator of cellular energy homeostasis [26–28]. Deleting SNF1 disrupts the cell’s ability to adapt to energy stress and creates a low ATP environment to study how compromised ATP generation influences proteostasis, and SNF1 deletion strains have reduced ATP levels [29]. Because aggregation-prone proteins often misfold under conditions of energy stress, the SNF1 deletion background provides a powerful model to dissect the mechanistic links between energy signaling, chaperone activity, and aggregate formation. WT and SNF1 mutant strains with a 3x integrated QUEEN reporter (MTY3264 and MTY3371 [30]) were purchased from the Yeast Genetic Resource Centre Japan (YGRC). The SNF1 mutant strain and the corresponding WT strain (BY4730) that were transformed with the Aβ42-GFP reporter were from the yeast deletion collection [31]. TZ was added to log phase cultures at 0–300 µM, with ATP evaluated 60 minutes later. TZ was added at 30-40 µM to Aβ42-GFP reporter strains following 3 hours of reporter induction with CuSO4. Sixty minutes after TZ addition, GFP fluorescence, biochemical ATP values, and protein expression were evaluated. 

#### Yeast microscopy and QUEEN ratiometric analysis

Bright field, differential interference contrast (DIC), and two fluorescence images (Fluorescein (FITC) (ex480 image) and Ex409 (ex409 image)) were taken from multiple fields through fluorescence microscopy. Open-source Fiji software was used to calculate the QUEEN ratio [32]. 

### Mice

#### 5xFAD mice

All experimental procedures were approved by the Institutional Animal Care and Use Committee (IACUC-Protocol #0062039) at the University of Iowa. 5xFAD transgenic mice (Tg(APPSwFlLon, PSEN1 *M146L*L286V)6799Vas) [33] and non-transgenic littermates were received from The Jackson Laboratory (Bar Harbor, ME). Mouse sample sizes of 10 per group (40 total) were selected based on a priori power analyses and followed from birth. Behavioral and histopathological analyses were unbalanced because of animal death between cohorts [20, 24].

#### Experimental design and timeline

5xFAD mice and non-transgenic littermates were randomized to water vs. TZ treatment at 5 weeks of age, followed by behavioral studies starting at 6 months of age. Mice were perfused after behavioral studies including interval timing assays and Barnes Maze assays, detailed as below. Tail plasma samples were collected before perfusion.

#### TZ administration

TZ (Tocris #1506, Minneapolis, MN) was delivered to mice through *ad lib* access to treated water in plastic water bottles, starting at 5 weeks of age and continuing until mice were euthanized. The vehicle control group had access to regular drinking water contained in the same type of plastic water bottles. Preparation of TZ began with a 41-mM stock solution (18.8 mg/ml of TZ HCl-dihydrate in water ~ 17.3 mg/ml of TZ HCl) stored at 4 °C for up to 1 week. We added 106 µL of stock solution, equating to 1.9 mg TZ HCl, to 300 ml water, to which mice had free access. The concentration of the final drinking water was 6.2 µg/ml TZ (1.9 mg/300 ml). An average mouse drinks ~ 5 ml of water per day [34], so each mouse consumed ~ 31 µg TZ daily (6.2 µg/ml x 5 ml/day). Additionally, experimental mice weigh on average 0.025 kg; thus, the TZ dose received by our animals was ~ 1.23 mg/kg/day (31 µg/day/0.025 kg). All water bottles containing TZ or water were replaced every 2 days for the duration of the experiment until mice were euthanized [20].

#### TZ dosing validation

Five C57/BL6 mice were treated with water vs. 5 C57/BL6 mice treated with TZ at ~ 1.23 mg/kg/day, for 3 weeks as described above. Water consumptions were monitored by weighing the water bottle daily. Mice were weighed daily. In this pilot study, the average rate of water consumption is 4.3 ml/mouse/day (average weight 23.0 ± 1.2 g, mean ± SEM) in the water group and 4.4 ml/mouse/day (average weight 22.0 ± 0.9 g) in the TZ group. The actual dose delivered is 1.27 mg/kg/day in the TZ group. Blood pressures were measured detailed as below. Mice were euthanized after 3 weeks of water vs. TZ treatment, with plasma samples and flash frozen brain samples collected for TZ concentration measurement.

#### Blood Pressure

To minimize stress-related variability and improve blood pressure signal quality, animals were systematically acclimatized to the tail-cuff procedure. Beginning prior to the recording day, animals were placed in the restraining cylinders for short, repeated sessions each day. These acclimatization sessions familiarized the animals with the restraint and maintained tail circulation under warm conditions, allowing stable, reproducible blood pressure signals to be obtained. By the third day, animals tolerated the restraint without excessive movement, enabling reliable measurement cycles. Systolic blood pressure, diastolic blood pressure, and pulse rate were measured non-invasively on Day 3 using a tail-cuff photoplethysmographic system (BP-2000 Blood Pressure Analysis System, Visitech Systems, USA). During each measurement session, animals were placed in ventilated restraining cylinders positioned on a warming platform to promote tail blood flow. Following a minimum of five preliminary conditioning cycles, 10–20 measurement cycles were recorded for each animal. All readings were automatically captured. Cycles exhibiting motion artifacts, unstable waveforms, or irregular pulse detection were excluded according to standard tail-cuff quality criteria. Valid cycles were aggregated for each animal.

#### Liquid chromatography-mass spectrometry (LC-MS) to measure mouse TZ plasma and brain concentration

The TZ assay was performed at the Metabolomics Core at the University of Iowa as described previously [35]. Briefly, 90 µl of plasma samples were mixed with 1300 µl of the chilled extraction solvent consisting of methanol, acetonitrile, and water in a 2:2:1 ratio (volume/volume/volume) containing 3.9 ng/ml of deuterium-labeled TZ. 10 µl of 100% water was added to the mixtures. For brain samples, 50 ~ 80 mg of flash frozen brain tissues were mixed with 800ul of the extraction solvent. Mixtures were vortexed for one minute and rotated at -20 °C for 1h to precipitate protein. Next, samples were centrifuged at 21,000 x g for 10 min at 4ᵒC to remove insoluble material. 1000 µl of supernatants (800ul in the case of brain samples) were transferred to clean microcentrifuge tubes and were evaporated to dryness using a Speed Vac and reconstituted in 25 µl of acetonitrile/water 1:1 ratio (volume/volume). Samples were stored at -20 °C for 2h and then centrifuged at 21,000 x g for 10 min at 4ᵒC. The supernatant was transferred to LC autosampler vials for analysis. We used mass spectrometry to quantify plasma TZ. LC-MS data were acquired on a Vanquish Flex UHPLC system (Thermo Scientific) coupled to a Thermo Q Exactive Hybrid Quadrupole-Orbitrap mass spectrometer. Two µL of metabolite extracts were separated using a Millipore SeQuant ZIC-pHILIC (2.1 × 150 mm, 5 μm particle size) column with a ZIC-pHILIC guard column (20 × 2.1 mm) attached to a Thermo Vanquish Flex UHPLC. Mobile phase was comprised of Solvent A: 20 mM ammonium carbonate, 0.1% ammonium hydroxide, and Solvent B: acetonitrile.

##### Barnes maze spatial learning and memory assay

At 8 months of age, mice were run on the Barnes maze assay in balanced cohorts using a 3-day optimized protocol for procedural learning and spatial memory acquisition [36]. Briefly, during the Barnes Maze assay, a mouse is placed on an elevated circular illuminated table with 10 ports arranged along the outer edge, one of which allows the mouse to escape to a preferred dark location—the target escape port. This protocol uses 3D-printed escape shuttles attached to the target escape port, combined with rotating the entire maze at each trial to reduce the possibility of unwanted cues interfering with spatial memory acquisition. On Day 1, mice performed one unanalyzed habituation trial and five spatial learning trials, with homecage bedding placed in the shuttle box at the bottom of all escape ports (target and non-target). The maze was turned every trial to minimize odor as a navigation cue. Intertrial intervals were 3 min from the time animals escaped. On Day 2, mice performed one probe trial to test early spatial retention ~ 24h after Day 1; on probe trials all ports were blocked to assess whether animals remember the location of the target escape port. Probe trials were followed by five spatial learning trials in which the escape port was unblocked. On Day 3, mice performed one probe trial to test spatial memory retention followed by five spatial learning trials. Data from the five spatial learning trials on each day were analyzed by averaging the distance travelled to reach the escape port. Data from the probe trials on Day 2 (“early”) and Day 3 (“late”) were analyzed as the percentage of time investigating the target spatial location, defined as a pie slice encompassing the target escape port and the two adjacent ports (3/10th of the maze) during a 90-second probe, and compared to a theoretical mean of 30%.

##### Switch interval timing assay

Our mouse-optimized switch interval timing task is designed to capture an animal’s internal representation of time as detailed in our prior work [37–43]. Mice are trained to switch from a “short” to a “long” nosepoke after approximately 6 s. This switch response is an explicit time-based decision that requires working memory for temporal rules and attention to the passage of time and our group and others have shown that this version of interval timing models cognitive deficits associated with neurodegenerative disease in mice [40, 44–46].

Briefly, mice were trained in standard operant chambers enclosed in sound-attenuated cabinets (MedAssociates, St. Albans, VT) that contained two light-equipped nosepoke response ports (left and right) on the front wall, a reward hopper (where food rewards are delivered) located between the two nosepoke response ports, and another light-equipped nosepoke on the back wall, opposite the reward hopper. Operant training began by “shaping” the animal’s behavior for 3 days. Trial initiation began with a response at the back nosepoke, at which point either the left or right front nosepoke was illuminated, and a response at the appropriate port resulted in a reward. After shaping, mice were then advanced to the interval timing switch protocol, for which each session was randomly organized into 50% short (6 seconds) and 50% long (18 seconds) trials. Either the left or right nosepoke was designated for short trials, and the contralateral nosepoke was designated for long trials (counterbalanced across mice and experimental groups). For both trial types, the trial is initiated with a back nosepoke response that generates two identical light cues above both the left and right nosepokes, along with an 8-kHz tone (72 dB). In short trials mice receive a reward after 6 seconds for the first response at the designated nosepoke. In long trials, mice begin by responding at the designated short-trial nosepoke but do not receive a reward. When there is no reinforcement after 6 seconds at the short-trial nosepoke, mice must switch to the designated long-trial nosepoke until a reward is delivered after 18 seconds. Median switch and CV times are plotted=. Only long switch trials were analyzed. Behavioral performance was analyzed for the last 4 days of training.

##### Histology

Mice were anesthetized with ketamine (100 mg/kg intraperitoneal (IP)) and xylazine (10 mg/kg IP) and transcardially perfused with cold PBS and 4% paraformaldehyde (PFA). Brains were removed and post-fixed in 4% PFA overnight, followed by immersion in a 30% sucrose in a PBS solution for at least 48h before 4–5-µm sections were collected and embedded in paraffin [40]. The Comparative Pathology Laboratory at the University of Iowa performed all sectioning and immunohistochemistry to visualize amyloid pathology in the mouse brain [47]. Antigen retrieval was done using citrate buffer (pH 6.0) in the decloaker (Biocare Medical, Pacheco, CA) at 110 °C for 15 min, followed by incubation in 6e10 monoclonal antibody (NBP2-62566, Novus, Centennial, CO) (1:5,000 in Dako diluent buffer) for 30 min at room temperature, and by incubation with the secondary antibody (anti-rabbit, HRP conjugated + Envision, Agilent) for 30 min. Sections were mounted, counterstained with hematoxylin for 3 min, and coverslipped. Slides were visualized using an Olympus BX53 microscope, DP73 digital camera, and CellSens Dimension Software (Olympus, Tokyo, Japan).

For amyloid pathology quantification, the pathologist (DM) examined tissues using the post-examination method of masking to group assignments [48]. The quantification was performed on stereologically matched sections sampled at fixed intervals along the rostro-caudal axis (every 120-µm coronal section, surrounding AP Bregma − 1.70 mm), and only sections falling within ± 0.24 mm of standardized anatomical reference levels were included in quantification. To assess in an unbiased approach, the slides were masked and examined for immunostaining using specialized software (Olympus BX53 microscope, DP73 camera, and CellSens Dimension Software). Areas of interest were defined as hippocampus (CA1 region) and cortex (immediately adjacent to the CA1 region). CA1 was selected because (i) it is among the earliest and most severely affected regions in human AD and in the 5xFAD model [33, 49, 50], (ii) CA1 pyramidal neurons are critically involved in the spatial memory tasks we used to assess behavior, providing a structural-functional link [51, 52], and (iii) prior 5xFAD characterizations have established robust amyloid burden in this region by 6 months of age [53, 54]. The adjacent cortical region (subiculum and overlying neocortex) was included because amyloid plaque deposition in 5xFAD is also prominent in these neighboring regions and they share vasculature and circuit connectivity with CA1 [55, 56].

These defined regions were evaluated with automated thresholding to detect 3,3-Diaminobenzidine (DAB) staining, and the output was the percentage (%) of the area of tissue that was positive. As a quality control for each slide, the pathologist confirmed the automated assessment thresholds targeted the amyloid plaques (i.e. brown spots) [57]. The amyloid staining (% area) data were analyzed using the Graphpad Prism Software (Graphpad, San Deigo, CA).

##### Mouse statistical analysis

We analyzed effects of TZ in amyloid pathology and interval timing using linear mixed effects models (Weber et al., 2023). Evaluation of learning rate for Barnes was analyzed with pre-planned comparisons at each day. Probe trials were tested for statistical evidence of learning by the group, by comparing the percent time spent in the target quadrant to a theoretical mean (30%) representing chance performance (Bertolli et al., 2024). Four sessions of interval timing behavior were collected and analyzed per mouse. All data and statistical approaches were reviewed by the Biostatistics, Epidemiology, and Research Design Core (BERD) at the Institute for Clinical and Translational Science (ICTS) at the University of Iowa. All code and data are made available at: http://narayanan.lab.uiowa.edu/article/datasets. An α of *p* < 0.05 was considered significant. Mice used in behavioral assays were also used for pathological analysis.

##### Statistics

We analyzed effects of TZ in amyloid pathology and interval timing using linear mixed effects models. Evaluation of learning rate for Barnes was analyzed with pre-planned comparisons at each day. Probe trials were tested for statistical evidence of learning by the group, by comparing the percent time spent in the target quadrant to a theoretical mean (30%) representing chance performance. All code and data are available at: http://narayanan.lab.uiowa.edu/article/datasets. As in our past work [24, 36, 58], analyses of mouse behavior were primarily done via analysis-of-variance (ANOVA; anova in R) of linear mixed effect models (lmer in R), which were selected because of their robustness in capturing both fixed and random effects, modeling mouse-specific variability, and accommodating unbalanced classes. Effect size was reported via Cohen’s d (cohen.d in R) for pairwise tests or f^2^ (eta_squared in R) for ANOVAs [59].

##### ADNI database analysis

The Alzheimer’s Disease Neuroimaging Initiative (ADNI) is a multi-site observational study of patients with mild cognitive impairment (MCI) or AD. Data from the ADNI database are made publicly available, and at the time of our analysis, there were 2,343 participants. The ADNI study collects extensive data, including drug use, clinical outcome measures related to AD disease progression, and multiple neuroimaging sequences, among others. Clinical outcomes include the Alzheimer’s Disease Assessment Scale-Cognitive 13 (ADAS-CoG 13; a measure of cognitive impairment, with higher scores indexing more advanced cognitive impairments) and the Clinical Dementia Rating -Sum of Boxes (CDR-SOB).

In addition to TZ, doxazosin (DZ) and alfuzosin (AZ) are also FDA approved quinazoline-based α1-antagonists that, like TZ, have been shown to bind and activate PGK1. We refer to these three drugs (TZ/DZ/AZ) collectively as “glycolysis-enhancing α1-antagonists” [20]. Meanwhile, Tamsulosin is also FDA-approved for benign prostatic hyperplasia and has the same indication and prescribing population as TZ/DZ/AZ, but is a sulfonamide-class α1-antagonist that does not bind PGK1 and does not enhance glycolysis [20]. This makes Tamsulosin an ideal active comparator, to control for confounding by the underlying indication (BPH and lower urinary tract symptoms) and by general α1-blockade effects, allowing us to isolate the glycolysis-enhancing activity as the variable of interest.

In the current study, we aimed to compare clinical and neuroimaging outcome measures between eligible ADNI participants who were using TZ/DZ/AZ, tamsulosin, or none of these drugs. To be eligible for inclusion in these analyses, participants had to be male (females using TZ/DZ/AZ were less common and more likely to use these drugs for other indications); have more than one eligible ADNI visit; and carry a diagnosis of MCI or AD at their first eligible ADNI visit.

Use of drugs of interest (TZ/DZ/AZ or tamsulosin) was defined as at least 30 days of use during the time that the participant was enrolled in the ADNI dataset. If the participant started and stopped one of the drugs of interest prior to entering the study, they were not considered to be using the drug. Most participants were already taking the drug of interest at the time that they entered the study; however, some began treatment during their participation in the ADNI study. In these instances, the first visit that the participant was known to be taking the drug of interest was defined as the baseline visit. Concomitant use of TZ/DZ/AZ and tamsulosin was prohibited for these analyses. If a participant switched from one drug class to another, only the time of their first eligible exposure was included, and subsequent visits after switching were discarded.

Given that male participants who would be prescribed TZ/DZ/AZ or tamsulosin were of similar age and had similar comorbidities, participants taking these drugs were grouped together. We then performed 2:1 propensity score matching using a nearest neighbor technique to match users of TZ/DZ/AZ or tamsulosin to participants who had never been exposed to either drug group. Matching was based on baseline values of age, ADAS-Cog13 scores, CDR-SOB, baseline diagnosis (MCI or AD), and the number of copies of the APOE4 allele, which is a key modifier of AD recorded in the ADNI database.

Most participants were included in the ADNI study for 5 or fewer years. After matching, we constructed linear mixed effects regression models to compare the trajectory of change of the CDR-SOB, the ADAS-Cog13, and the mean annualized change in FDG-PET standardized uptake value from composite ROI of AD-relevant areas, among participants taking TZ/DZ/AZ or tamsulosin and matched participants who used none of these drugs. The models included a per participant intercept, and the predictor of interest was the group*duration interaction. Specifically, “duration” was the number of years in the ADNI study for each participant.

All analyses were performed in RStudio version 4.2.2. Matching procedures were carried out using the *MatchIt* package (version 4.5.0), and LMER analyses were performed using the *lmerTest* package (version 3.1.3). Results were considered significant if the *p* value was < 0.05.

#### Merative Marketscan administrative data of insurance claims

##### Cohort construction

We used a new-user, active-comparator design. This is the gold standard design for observational pharmacoepidemiological investigations, as it reduces many important possible confounders, especially confounding by indication [60]. We identified all dispensing events for TZ, AZ, DZ, tamsulosin, finasteride, and dutasteride, and grouped these into three bins: TZ/DZ/AZ, tamsulosin, and finasteride/dutasteride. To ensure that we were identifying new users, we required at least 365 days of enrollment in the Merative Marketscan Commercial Claims and Encounters or Medicare Coordination of Benefits databases with prescription drug coverage before the first dispensing event. The first dispensing event was identified as our “index date,” and our “lookback period” was defined as the period before the index date, during which an individual was enrolled in an insurance plan contributing data to the Merative Marketscan database.

We defined AD status based on ICD-9-CM/ICD-10-CM codes. As with past work, we did not examine dose [21, 22, 24, 61]. We followed individuals from the first observed drug dispensing date (index date) until they were diagnosed with AD or left the Marketscan database, whichever happened first. If an individual left the Marketscan database for any reason, we censored their follow-up on the last observed enrollment date. The maximum follow-up period was 20 years (Table S3-5).

We then applied several exclusion criteria. First, the individual could not switch between the drug groups. Second, we required at least 1 day of follow-up after the index date. Third, these drugs are primarily used to treat BPH, with other indications being less common (TZ is used as a late-stage treatment for hypertension; tamsulosin can be used in the medical management of kidney stones). While most male individuals taking these drugs are likely using them for the management of BPH, no female users are managing BPH and were excluded. Excluding women reduces unobserved heterogeneity, although at the cost of some representativeness of our sample. Fourth, because AD and BPH are mostly diseases of aging, we removed anyone who was younger than 40 years old on the index date. Finally, we removed anyone diagnosed with AD on or before the index date.

##### Outcome definition

We defined an individual as having AD if they ever had an ICD-9-CM diagnosis code of 046.11, 046.19, 290.10, 290.11, 290.12, 290.13, 290.20, 290.21, 290.3, 290.40, 290.41, 290.42, 290.43, 291.2, 294.10, 294.11, 331.0, 331.11, 331.19, 331.2, 331.82; or an ICD-10-CM diagnosis code of F01.50, F01.51, F03.90, F03.91, F04, G30.0, G30.1, G30.8, G30.9, G31.1, G31.09, G31.1, G31.81, G31.82, G31.83, G31.84, G31.85, G31.89, G31.9, I69.90, I69.910, I69.911, I69.913, I69.914, I69.915, I69.918, I69.919, I69.920, I69.921, I69.922, I69.923, I69.928, I69.931, I69.932, I69.933, I69.934, I69.939, I69.941, I69.942, I69.943, I69.962, I69.963, I69.964, I69.965, I69.969, I69.990, I69.991, I69.992, I69.993, I69.998, R41.0, R41.1, R41.2, R41.3, R41.4, R41.81, R41.82, R41.83, R41,840, R41.842, R41.843, R41.844, R41.89, R41.9. These codes were based on prior published validations of these code sets to identify AD. For our outcome, we used the first observed date in any setting—outpatient, inpatient, or facilities claim—with one or more of these diagnosis codes. We created our case definition to identify the most possible cases of AD. However, many of these AD cases may have been misdiagnosed or assigned as a placeholder diagnosis during a longer neurological course of care. Thus, we performed a sensitivity analysis in which cases of AD were required to include at least two diagnoses of AD, with at least 365 days between the first observed and last observed diagnosis date. We reasoned that when an AD diagnosis recurs, and more specifically, recurs over a long time span, it is more likely that a person had AD, as opposed to another non-AD disorder. If a person was diagnosed with AD but did not meet this heightened case definition, we censored their follow-up on the first observed diagnosis date.

##### Merative statistical analysis

We followed individuals from the first drug dispensing date (index date) until they were diagnosed with AD or left the database. We used inverse probability of treatment weighting (IPTW), and we used entropy balancing to calculate IPTW weights [62]. We visualized differences in survival functions using the weighted Kaplan-Meier estimator, and estimated hazard ratios (HR) using weighted Cox proportional hazards regression. Kaplan-Meier weights were calculated using the R packages *ebal* and *WeightIt*; survival analysis was done using the R package *survival*; all analyses were done using R 4.1.

For further materials and methods, please see our Supplementary Materials and Methods.

## Results

### TZ increases ATP in yeast and mitigates amyloid beta (Aβ) 42 aggregation

**Fig. 1:**
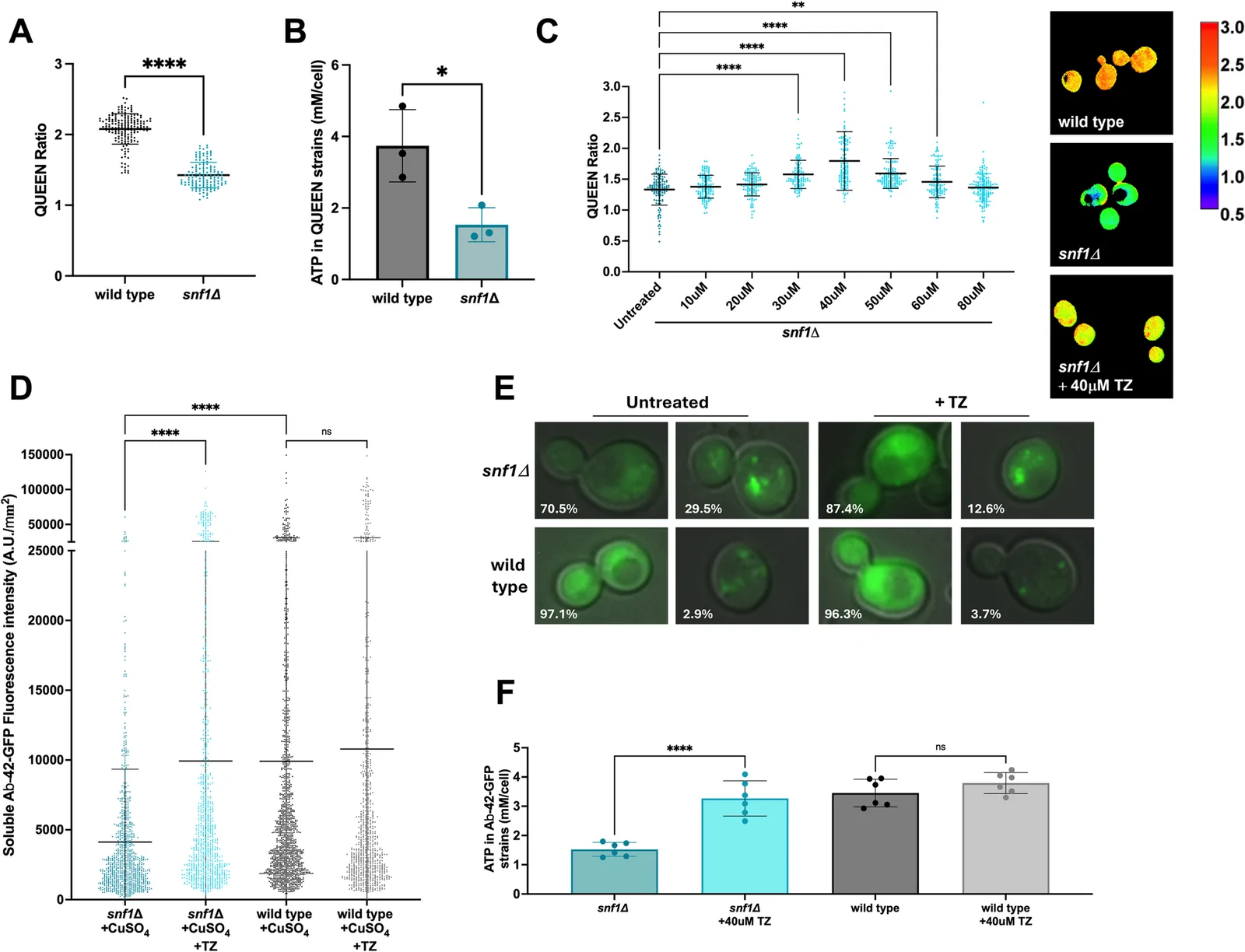
Terazosin (TZ) increases ATP levels and mitigates Aβ42-GFP aggregation in yeast. **A** Reduced ATP levels in the *snf1* mutant compared to wild-type yeast strains based on the QUEEN ATP biosensor. Each QUEEN data point represents the ratio within an individual cell; *n* = 500 cells per dose. **B** Luciferase-based biochemical measurement of cellular ATP levels conducted in triplicate (*p = 0.03) providing absolute intracellular ATP concentrations in the two strains. **C** ATP dose response curve for the *snf1* mutant after 1h exposure to TZ at the indicated concentrations and representative images of *snf1* mutant yeast cells colored to reflect the QUEEN ratio where blue/green values are assigned to lower QUEEN ratios (right). Error bars represent the mean and standard deviation. All comparisons between TZ treated samples and the 0 dose were significant at *p* < 0.02. D-E-F) The effect of TZ on ATP and puncta formation in cells expressing Aβ42-GFP. **D** Soluble Aβ42-GFP increases with TZ in *snf1* mutants. Data points represent soluble Aβ42-GFP fluorescence intensity of individual cells lacking puncta after a 4h CuSO_4_ induction and a 1h treatment with 40 µM TZ (note that the dose response curve for TZ in panel C shows a clear elevation in QUEEN signal at 40 µM). *n* > 800 cells per treatment category. Horizontal lines depict quartiles. **E** Representative images from D are shown with the percentage of cells exhibiting Aβ42-GFP puncta indicated. **F** 40 µM TZ increases ATP levels in the *snf1* mutant expressing Aβ42-GFP, based on luciferase-based biochemical measurement of cellular ATP. Significance for all panels was determined by ANOVA analysis with a post-hoc Tukey test (**** *p* < 0.0001; ns, non-significant)

We investigated the effects of TZ in yeast, a eukaryotic organism with a glycolytic pathway that is conserved in humans. We specifically focused on the yeast *snf1* mutant, which models impaired energy metabolism [29], and does not homeostatically regulate ATP as in wild-type cells [32], allowing for quantitative detection of ATP fluctuations which are otherwise challenging to capture in wild type. To assess ATP levels, we first implemented a single-cell approach using the integrated QUEEN (quantitative evaluator of cellular energy [63]) biosensor (Fig. 1A). This biosensor reports ATP-dependent fluorescence as a ratio of two excitation wavelengths, yielding a single-cell ratiometric measure of ATP-related changes that captures cell-to-cell variability within the population. To complement this measurement strategy, ATP was extracted from a defined number of cells originating from the same QUEEN-imaged cultures and quantified using a luciferase-based biochemical assay (Fig. 1B). Although this extraction method yields a population-averaged concentration and therefore cannot resolve heterogeneity among subpopulations (e.g. cells exhibiting markedly low ATP levels), it provides an absolute value (expressed as mM) that serves as a reference for interpreting the ratiometric output generated by the QUEEN biosensor. As expected, ATP levels were lower in *snf1* mutant yeast strains compared to wild type strains in both single-cell and population-level measurements (Fig. 1A–B; *p* = 0.0001 QUEEN; *p* = 0.03 luciferase) [64].

Treatment of the *snf1* mutant with TZ at concentrations between 30 and 60 µM resulted in a rapid increase in ATP levels(Fig. 1C) [20, 23, 65]. Of note, wild type strains of yeast homeostatically regulate ATP levels [32] and consequently ATP levels were not increased by TZ treatment (Fig S1A). Treatment of *snf1* mutant yeast with tamsulosin, an α_1_-adrenergic receptor antagonist that does not bind PGK1, did not elevate ATP levels (Fig S1B).

Next, we examined the effect of elevated ATP on the aggregation of Aβ42 in the *snf1* mutant expressing Aβ42-sfGFP (sf, superfolder) under the control of the yeast *CUP1* promoter [66], which can be induced by copper sulfate (CuSO_4_). This Aβ42-sfGFP construct is designed to give rise to a diffuse cytoplasmic signal when soluble that is reduced under conditions that promote Aβ42 aggregation [66]. First, we confirmed that neither the introduction of Aβ42-sfGFP expression plasmid, nor the induction by CuSO_4_ affected the reduced ATP level that we observed in *snf1* mutant yeast (Fig S1C). Since 40 µM TZ increased ATP (Fig. 1F), we next measured the impact of 40 µM TZ treatment on soluble Aβ42-GFP fluorescence in the *snf1* mutant following CuSO_4_ induction. The *snf1* mutant exhibited a very low level of soluble Aβ42-GFP fluorescence (indicating a high level of Aβ42 aggregation), which was significantly increased by TZ treatment (Fig. 1D). In addition, a substantial number of cells in the *snf1* mutant sample (29.5%) contained fluorescent puncta due to Aβ42-sfGFP aggregation, and the percentage of cells with fluorescent puncta was reduced following TZ treatment (12.6%) (Fig. 1E). The increase in soluble signal and reduction in cells with puncta following TZ treatment suggest that the reduced Aβ42-sfGFP aggregation may be associated with the increased levels of ATP. Of note, studies with the same design were performed in wild type yeast strains, and neither the soluble Aβ42-GFP fluorescence signals, nor the ATP levels were affected by TZ treatment (Fig. 1D and F). Critically, TZ treatment did not affect Aβ42-sfGFP expression levels (Fig S1D). In summary, these data suggest that TZ increased ATP levels in the yeast *snf1* mutant and TZ-mediated elevation in ATP may solubilize Aβ42 aggregates. To assess whether TZ modulates proteostasis under an independent mitochondrial stress paradigm, we used antimycin A to inhibit respiration and reduce ATP in wild-type cells. Under these conditions, TZ elevated ATP levels and altered Aβ42-sfGFP fluorescence, though the nature of this effect differed from that observed in the snf1Δ model, likely reflecting differences between acute mitochondrial inhibition and chronic ATP limitation, which bias the system toward distinct proteostatic states of Aβ42-sfGFP (Fig. S2).

### TZ improves neuropathology and cognitive function in the 5xFAD mouse model of AD

**Fig. 2:**
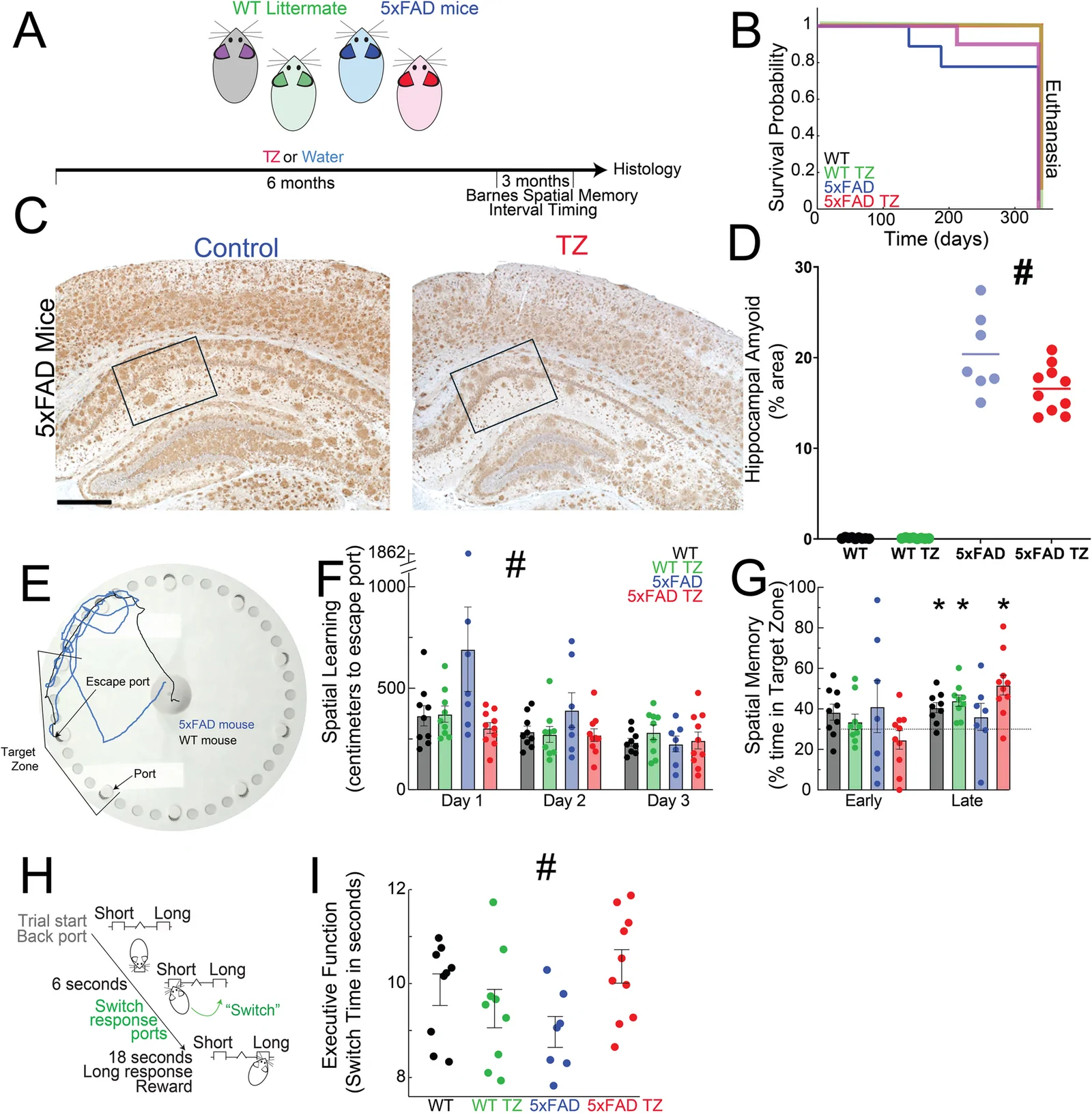
Terazosin (TZ) alleviates amyloid pathology and cognitive deficits in 5xFAD mice. **A** 5xFAD mice and littermate wild-type (WT) controls were treated with TZ or water for 6 months and then tested in behavioral assays. **B** Survival of 5xFAD mice vs. WT littermates treated with TZ vs. water. **C** We measured amyloid (% area) pathology in the hippocampal CA1 region using 6e10 antibody staining. Boxes approximate part of CA1 region, note the reduced amyloid plaques (brown foci) in the TZ group. **D** TZ decreased the amyloid (% area) burden in 5xFAD mice. Data from 17 5xFAD mice (10 receiving TZ and 7 receiving water) and 18 control mice (9 receiving TZ and 9 receiving water). **E** We measured spatial learning and memory using the Barnes maze assay in which mice learn which port allows them to escape from an elevated illuminated surface. During spatial learning trials, the distance travelled to the escape port is a measure of spatial learning. A single spatial learning trial from a 5xFAD mouse (blue) and a trial from WT mouse (black) are shown. **F** On Days 1–2, the distance travelled to the escape port was shorter in 5xFAD mice treated with TZ, compared to 5xFAD mice treated with water, suggesting that TZ improved the learning of this task. **G** We measured spatial memory by measuring time in the target zone (an area comprising the target and two adjacent ports) on probe trials when the escape port was blocked, early in training (on Day 2), and late in training (Day 3). Late in training, 5xFAD mice displayed poor spatial memory with less than > 30% in the target zone; this was rescued by TZ. **H** We tested executive function using a mouse-optimized switch interval timing task, in which mice estimate an interval of several seconds with a motor response (switch to another port) based on working memory for temporal rules as well as attention to the passage of time. **I** Switch responses, which measure executive function, were early in 5xFAD mice and rescued by TZ. Standard errors are shown on all plots; *p < 0.05 via paired t-tests; #*p* < 0.05 from an interaction between 5xFAD and TZ in linear mixed-effects models

We tested the hypothesis that enhancing glycolysis is neuroprotective against AD in the 5xFAD mouse model of AD. We selected the 5xFAD model because it contains five human AD mutations (three in the Aβ precursor protein (APP) gene, and two in the presenilin gene), and it has reliable amyloid aggregation at ~ 2 months of age and deficits in cognitive function assays by ~ 5 months. We started TZ treatment (1.2 mg/kg/day in drinking water) in 5xFAD mice at ~ 5 weeks of age (Fig S3). All mice were treated for ~ 5 months prior to performing behavioral testing (Fig. 2A). All 10 5xFAD mice treated with TZ survived to 11 months, when they were perfused for histology. Three 5xFAD mice (2 males, 1 female; 7 surviving) receiving water died before 11 months; 1 WT male mouse receiving water and 1 WT female mouse receiving TZ died before 11 months (9 surviving in each category). A Cox proportional model revealed no reliable differences in survival between 5xFAD mice receiving TZ or water (*p* = 0.52; Fig. 2B). 35 mice were available for subsequent analyses.

In 5xFAD mice treated with TZ, we examined Aβ pathology through immunohistochemistry using an established amyloid (% area) scoring scale, quantified by a veterinary pathologist who was blinded to treatment condition [48]. The amyloid (% area) plaques in the CA1 region of the hippocampus was significantly lower in 5xFAD transgenic mice treated with TZ vs. water (main effect of TZ in 5xFAD mice; *p* = 0.04, f^2^ = 0.34; Fig. 2C–D). No differences in amyloid scores were observed in the cortex (Fig S4).

We investigated whether TZ improved cognitive performance in 5xFAD mice. First, we performed the Barnes Maze assay of spatial learning and memory [36]. On each spatial learning trial, mice are placed on a circular, illuminated table with 10 ports arranged along the outer edge, one of which allows the mouse to escape to a preferred dark location—the target escape port. As a measure of spatial learning, we quantified how far mice travelled from the center of the table to the target escape port. A quicker reduction of total distance travelled is associated with faster spatial learning (Fig. 2E). While all mice progressively decreased their distance travelled over 3 days of training suggestive of learning through spatial memory consolidation, 5xFAD mice consistently travelled longer distance compared to other groups, indicating slower learning (*p* = 0.04, f^2^ = 0.30) [36]. Strikingly, TZ compensated for spatial learning deficits in 5xFAD mice (no main effect of 5xFAD mice; main effect of TZ in 5xFAD mice: *p* = 0.04, f^2^ = 0.14; interaction between 5xFAD mice and TZ across all days: *p* = 0.04, f^2^ = 0.15; Fig. 2F).

We also quantified spatial memory via performance on probe trials, during which the target escape port is blocked [36]. Early in training, there was no difference between the percentage of time that all mice spent in the target zone over the blocked escape port (including the escape port and two flanking ports; 3/10 ports, or 30% chance; Fig. 2G), implying that early Barnes Maze training was insufficient to consolidate spatial memory. However, later in training, WT mice treated with water and with TZ spent significantly more time in the target sector compared to chance (*p* = 0.003, Cohen’s d = 1.4; and *p* = 0.001, Cohen’s d = 1.7, respectively). 5xFAD mice exhibited impaired spatial memory, performing no better than chance (*p* = 0.34); however, 5xFAD mice treated with TZ spent significantly more time in the target sector compared to chance (*p* = 0.003; Cohen’s d = 1.4). Of note, TZ did not affect total distance travelled during probe trials (Fig S5). Also, there was no correlation between total distance travelled during probe trials and distance travelled to the escape port during spatial learning trials (*r*=-0.14, *p* = 0.41). These data indicate that TZ was associated with improved spatial learning and memory in 5xFAD mice.

Finally, to investigate the effect of TZ on executive function, we used an interval timing assay that required mice to estimate an interval of several seconds by making a motor response—switching from one nosepoke response port to another. This assay requires working memory for temporal rules and attention to the passage of time [67]. In this mouse-optimized version, the timing of when mice switch from one nosepoke response port to the other reflects internal estimates of time [24]. Interval timing is impaired in humans with AD as well as 5xFAD mice, which tend to have anticipatory switch times, or earlier switch times, compared to control mice [68]. Strikingly, TZ compensated for 5xFAD deficits in interval timing (interaction between 5xFAD mice and TZ on switch time: *p* = 0.02, f^2^ = 0.19; Fig. 2I).

To further investigate TZ target engagement, we performed untargeted GC-MS plasma metabolomics and found that TZ produces a coordinated, 5xFAD-specific perturbation of plasma glycolytic intermediates. Pathway-level evidence was robust (within-genotype permutation test *p* = 0.0013; MANOVA *p* = 0.014 across the pre-specified 10-metabolite glycolysis panel), with 3-phosphoglycerate, the direct product of the PGK1 reaction, increased 1.88 fold after TZ treatment in 5xFAD mice (surviving panel Bonferroni correction *p* = 0.024; Fig S6).

Water intake, body weight, were comparable between TZ- and water-treated animals in both genotypes (Table S1-2). Calculated TZ exposure was 1.2 mg/kg/day, comparable on an allometric basis to the human BPH dose, with quantifiable plasma and brain drug concentrations confirmed in a pilot cohort. Tail-cuff systolic blood pressure was not significantly changed between water and TZ treated groups in a pilot cohort of mice. Together, these data exclude gross health, motor, or cardiovascular confounds as drivers of the observed cognitive improvements. Taken together, these data suggest that TZ is protective in animal AD models.

### TZ is associated with a slower decline of cognitive function and FDG-PET biomarkers

To examine if these changes in mice were linked with human AD, we analyzed the Alzheimer’s Disease Neuroimaging Initiative (ADNI), which collected sequential neuroimaging and clinical data from a cohort of patients with AD [69], hence providing a unique resource to test whether TZ has neuroprotective potential in human AD. We found 61ADNI participants who were taking either TZ or two related drugs that also enhance PGK1 activity (doxazosin (DZ) or alfuzosin (AZ)) and 92 participants who were taking tamsulosin, an α_1_-adrenergic receptor antagonist that does not bind to PGK1. 153 participants taking either TZ, DZ, AZ (TZ/DZ/AZ) or tamsulosin were matched to 306 participants who were not using any of these drugs (non-users; controls) (Table S3-4). We compared TZ/DZ/AZ users to tamsulosin users and non-user controls.

**Fig. 3:**
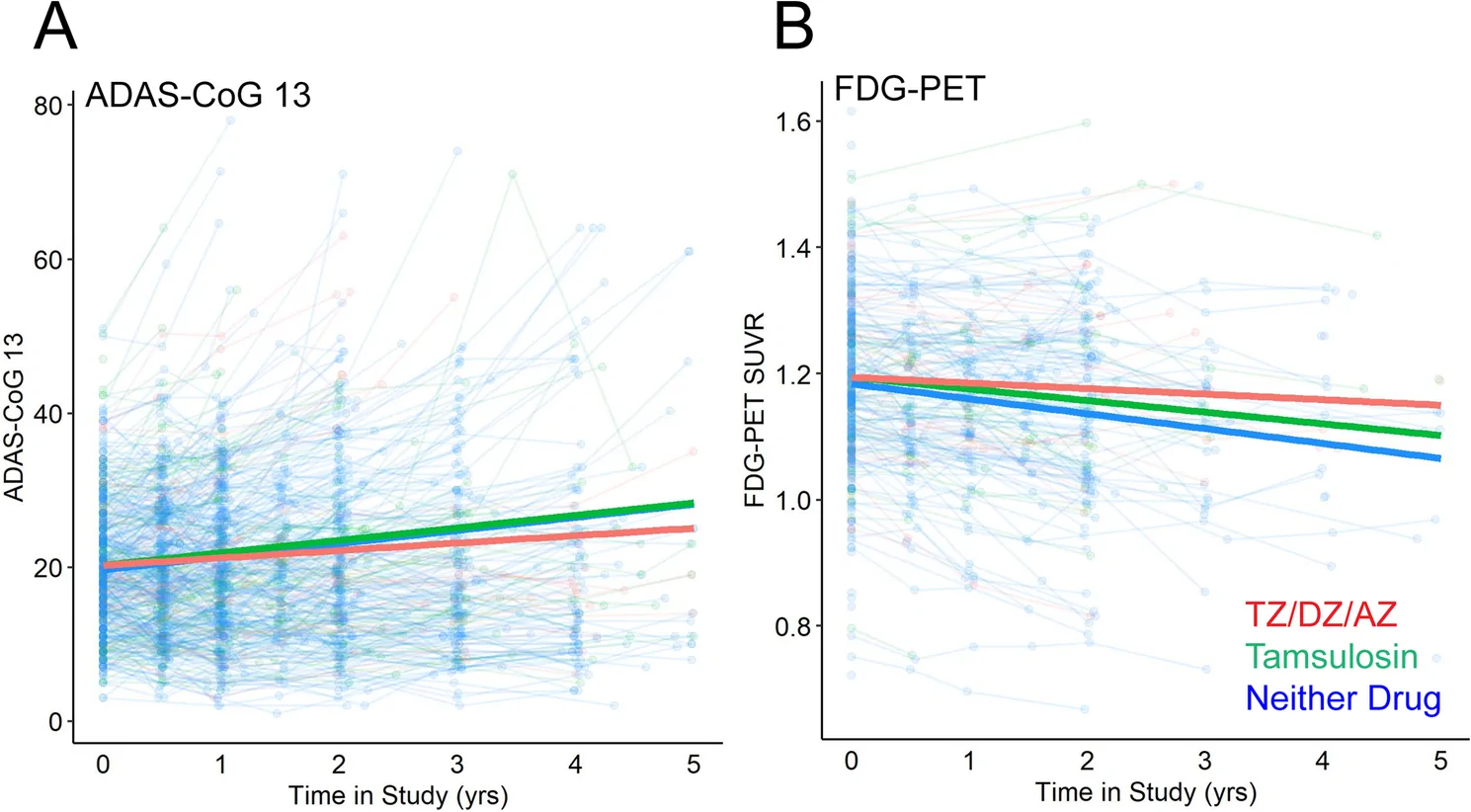
Patients with Alzheimer’s disease taking TZ/DZ/AZ had slower cognitive decline and slower progression of ^18^F-FDG-PET. We analyzed the Alzheimer’s Disease Neuroimaging Initiative (ADNI) database and found slower cognitive decline (**A**), as indicated by an increase in the ADAS-Cog 13 score, and less decrease in ^18^F-FDG-PET (**B**) in AD patients taking TZ/DZ/AZ compared with AD patients taking tamsulosin or none of these drugs; note that fewer patients had PET scans later in the study

We found that the mean annualized rate of change of cognitive function as measured by the Alzheimer’s Disease Assessment Scale-Cognitive 13 (ADAS-CoG 13; a measure of cognitive impairment, with higher scores indexing more advanced cognitive impairments) was 1.71 points per year (ppy) (95% CI [1.48–1.93]) in patients taking neither drug, which was not significantly different from patients taking tamsulosin (1.62 ppy (95% CI [1.16–2.08]; *p* = 0.74)). Remarkably, the TZ/DZ/AZ group had a slower mean annualized rate of change of the ADAS-CoG 13 (0.96 ppy, 95% CI [0.42–1.51]; *p* = 0.01) compared to the non-user group (Fig. 3A), which would be clinically significant at ~ 2 years [70]. The rate of progression of the ADAS-CoG 13 was also slower in the TZ/DZ/AZ group relative to the tamsulosin group, but the results did not reach statistical significance (*p* = 0.07).

The ADNI database also includes extensive neuroimaging biomarkers, including a measure of brain metabolism, ^18^F-FDG-PET [4]. In line with a slower progression of cognitive impairment, we found that the rate of decline in the FDG-PET signal was significantly slower in the TZ/DZ/AZ group (mean annualized change = -0.009, 95% CI [-0.019–0.002]; (*p* = 0.012)) compared to non-user controls (mean annualized change = -0.02, 95% CI [-0.028 – -0.019]; Fig. 3B). The tamsulosin group did not reliably differ compared to the other two groups (mean annualized change = -0.02, 95% CI [-0.029 – -0.008]). These data provide additional evidence that glycolysis-enhancing drugs might be protective in AD.

### TZ/DZ/AZ is associated with a lower AD risk

We investigated a large-scale administrative insurance claims database to explore whether TZ is associated with a lower risk of AD. Using the Merative Marketscan databases of insurance claims, we identified 1,028,561 users of TZ, DZ, or AZ (TZ/DZ/AZ); 2,854,989 users of tamsulosin; and 653,508 users of 5-α-reductase inhibitors (5ARIs~ finasteride, dutasteride), another class of drugs for benign prostatic hyperplasia (BPH). We used an active-comparator and new-user design, the gold standard in observational pharmacoepidemiological studies [60], which has not been reliably used in retrospective studies of BPH drugs and dementia [61, 71, 72]. After applying exclusion criteria, our final cohort included 209,146 new users of TZ/DZ/AZ, 1,013,108 new users of tamsulosin, and 120,431 new users of the 5ARIs (Table S5).

**Fig. 4:**
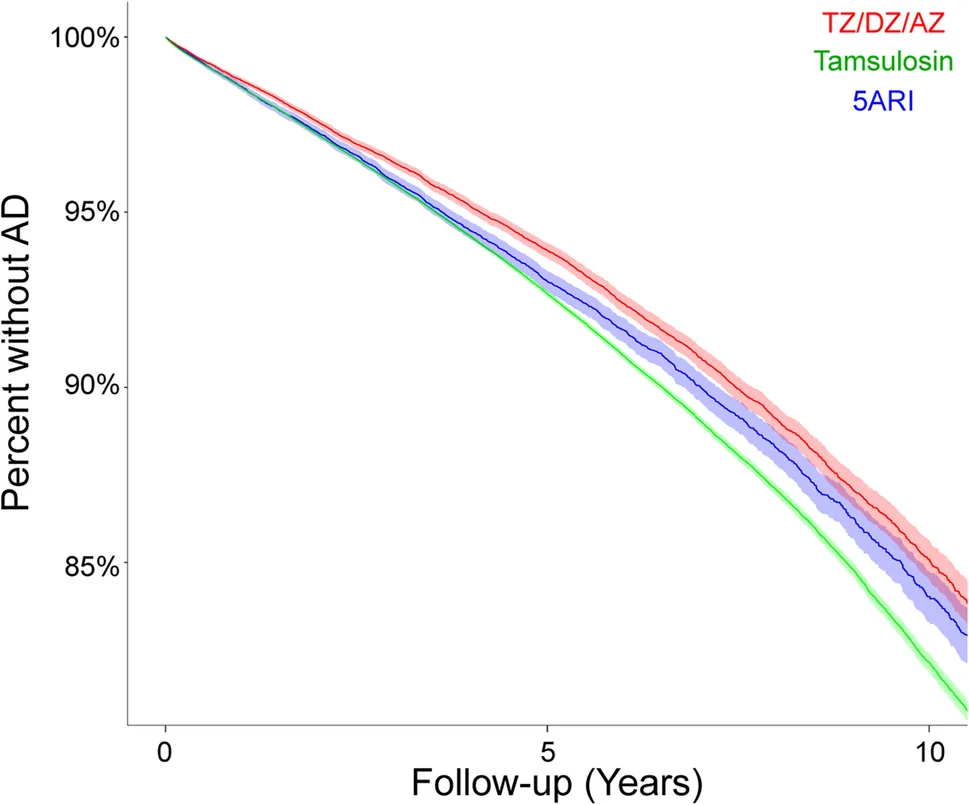
Patients taking TZ/DZ/AZ have less risk of Alzheimer’s disease (AD) in the Merative Marketscan human database of insurance claims. A Kaplan-Meier curve of the probability of AD diagnoses calculated from the Merative Marketscan dataset for users of TZ/DZ/AZ (red) vs. tamulosin (green) or 5ARI (blue). Patients using TZ/DZ/AZ were *less likely* to get AD than patients using tamsulosin or 5ARI

New users of TZ/DZ/AZ were followed for 594,862 person-years, and we observed 7,054 new cases of AD (11.9 cases per 1,000 people per year). This rate was lower than the rates observed among new users of tamsulosin (2,761,034 person-years; 46,915 cases; 17.0 cases per 1,000 people per year) or 5ARI (352,377 person-years; 4,586 cases; 13.0 cases per 1,000 people per year). Correspondingly, there was greater AD-free survival among TZ/DZ/AZ users compared to either tamsulosin or 5ARI users (Fig. 4). The estimated hazard ratio for AD for TZ/DZ/AZ users was lower compared to tamsulosin users (HR = 0.69, t = -29.4, *p* < 10^− 16^, 95% CI: [0.67–0.71]). Critically, the hazard of AD was also lower for TZ/DZ/AZ users compared to 5ARI users (HR = 0.77, t = -18.2, *p* < 10^− 16^, 95% CI: [0.74–0.88]) demonstrating that these results could not be readily explained by effects of tamsulosin alone [22].

There were some notable differences between the groups at baseline (Table S6). For instance, users of TZ/DZ/AZ were much more likely to have hypertension, renal disease, and diabetes with complications. These differences were mitigated through our weighting algorithm and propensity-score matching (Table S7). The lookback period and follow-up durations, which were not included in the inverse probability of treatment weighting (IPTW) adjustment, were similar across all three groups.

The association between a lower hazard of AD and the use of TZ/DZ/AZ persisted after adjustment. In a weighted pseudo-population, we observed 7,200 cases of AD in 533,753 person-years of follow-up among new users of TZ/DZ/AZ (13.5 per 1,000 people per year). Users of 5ARI (322,522 person-years, 4,887 cases, 15.2 cases per 1,000 people per year) and users of tamsulosin (2,870,393 person-years, 47,228 cases, 16.5 cases per 1,000 people per year) had higher incidence rates. The survival curve (Fig. 4) shows lower AD incidence after adjustment through IPTW. The Cox proportional hazards model shows a lower hazard of AD in new users of TZ/DZ/AZ compared to new users of tamsulosin (HR = 0.84, t = -867.9, *p* < 10^− 16^, 95% CI: [0.84–0.84]), and a smaller difference between users of 5ARI and tamsulosin (HR = 0.93, t = -385.5, *p* < 10^− 16^, 95% CI: [0.93–0.93]).

Inspection of the Schoenfeld residuals indicated violations of the proportional hazards assumption. We re-estimated our model with time-varying coefficients (Table S8). Across all time intervals, compared to users of tamsulosin, users of TZ/DZ/AZ had a reduced hazard of AD (HR of 0.80–0.85) that was consistent with the overall HR estimate (0.84). After inclusion of the time-varying coefficients, the Schoenfeld residuals indicated no violations of the proportional hazards assumption (*p* = 0.61).

As a negative control, we identified 212,887 new users of TZ/DZ/AZ, 1,043,753 new users of tamsulosin, and 123,094 new users of 5ARIs who were free of any malignant neoplasm of the brain at medication start. There were 487 cases of malignant neoplasm among users of TZ/DZ/AZ (incidence per 10,000 people per year: 7.95), 2,251 cases among users of tamsulosin (incidence per 10,000 people per year: 7.80), and 233 cases among users of 5ARI (incidence per 10,000 people per year: 6.39). Kaplan-Meier survival curves show no evidence of clinically meaningful differences between the groups (Fig S7), a finding supported by the Cox proportional hazards regression analysis (TZ/DZ/AZ versus tamsulosin HR = 1.03; 95% CI: 1.03, 1.03; 5ARI versus tamsulosin HR = 0.86; 95% CI: 0.86, 0.86).

Finally, we performed additional sensitivity analyses (Table S9). The estimated associations were effectively unchanged when patients with a neurological history were excluded, or a 1-year delay in follow-up was implemented. Also, E-values for unmeasured confounding and selection bias were calculated (Table S10). Additionally, we found a dose-response-like association between TZ/DZ/AZ and AD hazard, with longer durations of TZ/DZ/AZ exposure being associated with lower hazard of AD compared to IPTW-adjusted users of tamsulosin (Table S11). Taken together, these data demonstrate that TZ/DZ/AZ may be protective against the development of AD when compared to both tamsulosin and 5ARI, and imply that TZ/DZ/AZ may be protective in AD.

## Discussion

We tested the hypothesis that enhancing glycolysis is potentially protective in AD. We found that the glycolysis-enhancing drug TZ (1) increased ATP and decreased Aβ aggregation in one yeast model and altered the proteostatic state of Aβ in a second yeast model, (2) decreased hippocampal Aβ pathology in 5xFAD mice and protected behavioral performance of 5xFAD mice in the Barnes Maze assay of spatial memory, and in the switch interval timing assay, (3) decreased rates of cognitive decline and slowed the progression of FDG-PET imaging abnormality of participants in the ADNI dataset, and (4) is associated with a lower risk of developing AD in the Merative Marketscan human dataset of insurance claims. These data provide convergent evidence that enhancing glycolysis with TZ has therapeutic potentials in AD. While testing the efficacy of glycolysis-enhancing drugs such as TZ will require rigorous clinical trials, our data provide evidence of a novel mechanism of neuroprotection that is relevant for AD.

AD presents a significant public health challenge, with energy metabolism impairment recognized as a key pathological feature contributing to its progression [4–6]. In recent years, the exploration of TZ, primarily used as an α_1_-adrenergic receptor antagonist in hypertension and BPH, has revealed protective properties beyond its traditional indications. TZ binds to PGK1 and increases ATP in cell lines, in flies, and in preclinical mouse models [20, 73, 74]. TZ augments cellular energy metabolism, which may be crucial in mitigating neurodegenerative processes through a variety of mechanisms [20–23, 25, 73, 75]. Our ability to study this relationship in yeast is exciting because it might identify fundamental principles of how ATP interacts with protein aggregation, which will lead to a further understanding of pathological mechanisms and interventions [75, 76]. The *snf1* mutant provides a powerful mechanistic model of energy sensing failure and ATP-stress that is conceptually linked to neurodegenerative pathophysiology. Combined with previous reports showing that ATP levels in brain tissue of AD transgenic mice are reduced [77] and data supporting brain glucose hypometabolism and bioenergetic stress in human AD [78–80], the impact of TZ on Aβ42 aggregation in yeast provides groundwork and motivation for investigating glycolysis enhancing drugs as novel AD treatments.

This work is supported by a recent report demonstrating that TZ was neuroprotective in another AD mouse model [25]; although our study is distinct in examining yeast, the ADNI database, and retrospective analyses with active comparators. This prior study found effects while manipulating the α_1_-adrenergic receptor and interpreted their data in this context. Our past work has shown that TZ interacts directly with PGK1, independent of its α1-adrenergic receptor effects, and that other drugs with α1-adrenergic receptor antagonism do not interact with PGK1, do not increase cellular ATP, and are not neuroprotective [20, 24]. Our data extend to real-world human observational data from ADNI and Merative Marketscan databases to investigate the clinical relevance of TZ in human AD. The ADNI dataset provided valuable insights into the longitudinal effects of TZ on the progression of AD. Participants with AD who were treated with TZ/DZ/AZ exhibited a slower progression of cognitive dysfunction measured by ADAS-Cog 13 scores and a slower decline in FDG-PET signal, indicative of preserved neuronal metabolic activity. These findings suggest that TZ may not only slow cognitive decline but also preserve functional brain activity associated with AD pathology. Analyses of the Merative Marketscan database revealed a lower hazard of developing AD among patients prescribed either TZ or a related glycolysis-enhancing drug (AZ or DZ) compared to those prescribed tamsulosin, an α1-adrenergic receptor antagonist, and 5ARI without glycolysis-enhancing properties. This retrospective cohort analysis underscores the potential clinical benefit of glycolysis-enhancing drugs in reducing AD risk. Our work is supported by a recent study suggesting that TZ was associated with a lower risk of dementia compared to tamsulosin [71]. While it is possible that tamsulosin worsens AD, protective effects of TZ in our AD preclinical models, in PD preclinical models, and in retrospective analyses with active comparators [20–22, 72, 81–84], argue against tamsulosin worsening neurodegenerative diseases. This inclusion of active comparisons is critical in our analysis, as patients not taking any drugs for BPH are categorically different from patients who take drugs for BPH (suggesting that they have routine access to healthcare). Our current analysis of TZ/DZ/AZ in AD accounts for these factors [21, 22].

In the Merative Marketscan human database, our results found an association between the use of TZ/DZ/AZ and a lower risk of AD, compared to the use of either tamsulosin or 5ARIs. This effect persisted and was largely unchanged in several sensitivity analyses using alternative case definitions, delayed follow-up starts, or important sub-groups of interest. The effect of the use of TZ/DZ/AZ is temporally consistent with time-varying coefficients, showing a hazard ratio between 0.80 and 0.85 for 10 years after the medication start date, compared to that seen with tamsulosin use. Interestingly, in two of the sensitivity analyses, the results pointed toward a plausible causal interpretation of these results. First, we observed a slightly lower hazard of AD diagnosis for users of 5ARI compared to tamsulosin. However, it became clear that the association between 5ARI use and AD hazard was time-varying (Table S8). The time-varying coefficients show no difference in the hazard of AD between users of 5ARI and users of tamsulosin in the first 3 years following the drug start date. It seems unlikely that a causal effect of 5ARI on AD risk would only manifest 3 years after treatment start. However, use of TZ/DZ/AZ is consistently associated with a lower hazard of AD at all time points. Second, we found a muted effect of TZ/DZ/AZ in a subgroup with neurological comorbidities, which could include patients on a path toward an AD diagnosis (Table S9). The incidence rate of AD in this subgroup of patients is much higher than that of people without neurological comorbidities (~ 65 per 1,000 people per year compared to ~ 13 per 1,000 people in our data). Furthermore, as expected we found no difference between the TZ/DZ/AZ and tamsulosin groups for other diseases, such as brain cancer.

The metabolic engagement of TZ invites consideration of how TZ-induced glycolytic effects might interact with AMPK and downstream signaling pathways effects [85, 86]. The direction of TZ’s effect on AMPK is unclear: because TZ activates PGK1, the first ATP-generating step of glycolysis [75, 87], sustained ATP output should *reduce* AMPK activation by lowering the AMP/ATP ratio, while the bioenergetic stress characteristic of AD may dominate the equilibrium differently. The AMPK–AD relationship is not unidirectional [16]. AMPK activation has been linked both to beneficial effects on Aβ clearance [88, 89] and to pathological tau phosphorylation [15, 88] and autophagy-mediated synapse loss [90]. Recent work indicates that AMPK activation does not enhance autophagy in neurons in the way it does in non-neuronal cells; only direct mTORC1 inhibition reduces Aβ in APP/PSEN1 mice [91]. Our findings could suggest that TZ produces a disease-context-specific metabolic footprint, with mechanistic downstream consequences yet to be established. Direct measurement of phospho-AMPK (Thr172), phospho-S6K1, LC3-II/I, p62/SQSTM1, and Aβ degradation kinetics in TZ-treated 5xFAD brain — not performed here — are identified as priority follow-up experiments.

Although we focus on amyloid burden and glycolytic metabolism in 5xFAD mice, AD pathophysiology in humans is multifactorial. Several additional pathways warrant brief consideration. (1) Tau pathology: 5xFAD does not robustly develop neurofibrillary tangles. Whether glycolytic enhancement modifies tau pathology must be tested in a tau-positive model; AMPK is a known tau kinase [15, 92] and the net effect of bioenergetic enhancement on tau is yet unclear. (2) Neuroinflammation: AD microglia show a pro-inflammatory glycolytic shift, and Pan et al. [93] demonstrated that an aberrant glycolysis/H4K12-lactylation/PKM2 loop in plaque-adjacent microglia drives 5xFAD pathology. This appears to conflict with the present findings; we propose the resolution lies in cell-type and target specificity, with TZ acting at neuronal PGK1 — distinct from the PKM2/HK2 nodes driving the microglial loop. Direct microglial measures (Iba1, DAM markers, H4K12la, PKM2) in TZ-treated brain are an essential next step. (3) Insulin resistance, mitochondrial function, and synaptic biology: TZ acts downstream of insulin signaling and could in principle complement upstream metabolic interventions such as GLP-1 agonists [17, 19]. Finally, synapses are the most energetically demanding neuronal compartment [94] and synaptic loss is the strongest pathological correlate of cognitive decline [95]. The present study does not directly test these alternative pathways, and we frame them as opportunities for follow-up rather than established components of the mechanism.

Our results have several limitations. First, there are many mouse models that capture aspects of human AD, and our work supports prior findings in the APP/Swe model [25]. However, our work involves smaller sample sizes, which may limit generalizability. Also, we included equal numbers of male and female mice, but our study was not powered to detect sex differences, as this was not the main focus of our current study. Second, although plasma metabolomics showed strong TZ target engagement, we did not have data for direct PGK1 target engagement in brain tissue, glycolytic enzyme expression changes, and direct ATP measurements. However, TZ did increase ATP levels in our yeast models. Due to differences in phylogenetics and methodology, it is difficult to compare some details between yeast, mice and humans, but it is impressive that TZ reliably increases ATP in yeast, as has been previously reported in human cell lines, mouse brain, and humans [20, 23, 75]. In addition, future studies would benefit from including assays such as total Aβ/APP-related species, soluble and/or insoluble Aβ, total tau, phospho-tau, and synaptic or neuronal injury markers. Third, we report that in the ADNI database, compared to tamsulosin, TZ/DZ/AZ nonsignificantly improved the CDR-SOB and slowed AD progression as measured by FDG-PET, although these differences were not statistically significant. It is compelling that the TZ/DZ/AZ group is distinct from controls in the ADAS-Cog13 and FDG-PET, and that it provides a hypothesis-driven signal in a well-described AD cohort. Our analysis has several limitations inherent to ADNI, including small sample sizes, a limited ability to control for confounders, and sparse follow-up, particularly of FDG-PET. Furthermore, the retrospective nature of our Merative Marketscan database analyses, and inherent biases in ADNI, may influence the interpretation of our results, as there may be bias in prescriptions of tamsulosin in one group relative to another. We used a rigorous analytical framework with the active-comparator and new-user design to further reduce confounding by IPTW and included two active comparators. However, our ability to control confounding and obtain valid estimates depends on the accuracy and completeness of data observed. Providers may have important considerations when prescribing TZ/DZ/AZ vs. tamsulosin, such as hypotension or general frailty, that are correlated with AD risk but are not recorded in the claims record. Indeed, despite these drugs being primarily used for the treatment of BPH, most users do not carry the diagnosis of BPH. It is possible that important confounders are omitted, incompletely recorded, or otherwise unknown. Although we included alternative case definitions, delayed follow-up, and subgroup analyses, and we observed consistent results across all these analyses, the risk of omitted confounders remains. We do not have detailed clinical data on these patients included in our analyses, such as MoCA scores, which may be a more sensitive measure of neuroprotection and progression. Furthermore, our retrospective analyses primarily include men, which limits the generalizability of results, particularly because women bear a greater burden from AD [96]. Despite our efforts, it is possible that there is some residual confounding. While some of these concerns might be addressed with more advanced retrospective analyses, they can only definitively be addressed with a prospective randomized double-blind clinical trial; the numbers and design of this trial would be necessarily large and costly.

## Conclusion

In summary, we provide evidence that glycolysis-enhancing drugs increase ATP and decrease amyloid aggregation in yeast mutants. They also mitigate amyloid aggregation and preserve cognitive behaviors in the 5xFAD mouse model of AD. Furthermore, in ADNI we find slower progression of the ADAS-Cog13 and FDG-PET in patients taking glycolysis-enhancing drugs; in the Merative Marketscan database, we find that patients taking these drugs have decreased risk of being diagnosed with AD. Although observational human data and preclinical rescue do not constitute proof of clinical efficacy, our results suggest enhancing glycolysis as a potential therapeutic strategy in AD, and could inspire future clinical trials of repurposed glycolysis-enhancing drugs and new research into novel AD-relevant mechanisms.

## Supplementary information

Supplementary Material 1.

## Data Availability

All code and raw data are available at [https://narayanan.lab.uiowa.edu](https:/narayanan.lab.uiowa.edu).
