# Supplementary material for: Glycolysis-enhancing α1-adrenergic antagonists have therapeutic potential in Alzheimer’s disease
Source: Alzheimers Res Ther. 2026 Jul 9;18:222. doi: 10.1186/s13195-026-02135-z (PMC13644072; doi:10.1186/s13195-026-02135-z)
Supplement: Supplementary file 1 [file 13195_2026_2135_MOESM1_ESM.docx]

**Glycolysis-enhancing α_1_-adrenergic antagonists have therapeutic potential in Alzheimer’s disease**

Qiang Zhang*^1^, Jordan L Schultz^2^, Jacob E Simmering^3,^ Braedon Q Kirkpatrick^1^, Matthew A Weber^1^, Sydney Skuodas^4^, Tara Hicks^4^, Grace Pierce^4^, Margaret Laughlin^4^, Benu George^1^, Aimee X Bertolli^1^, Travis Larson^5^, Ramasamy Thangavel^1^, Mayu Oya^6^, David Meyerholz^7^, Georgina Aldridge^1^, Jan Fassler^4^, Nandakumar S. Narayanan*^1^

**Affiliations:**

^1^ Department of Neurology, Roy J. and Lucille A. Carver College of Medicine, University of Iowa, Iowa City, IA 52242.

^2^Department of Psychiatry, Roy J. and Lucille A. Carver College of Medicine, University of Iowa, Iowa City, IA 52242.

^3^ Department of Internal Medicine, Roy J. and Lucille A. Carver College of Medicine, University of Iowa, Iowa City, IA 52242.

^4^ Department of Biology, University of Iowa, Iowa City, IA 52242.

^5^ Duke Medical Scientist Training Program and Department of Neurobiology, Duke University, Durham, NC 27710.

^6^ Johns Hopkins University, Baltimore, MD, 21205.

^7^ Department of Pathology, University of Iowa, Iowa City, IA 52242.

**Conflict of Interest:** There are no conflicts of interest.

***Correspondence to:**

[Qiang-zhang@uiowa.edu](mailto:Qiang-zhang@uiowa.edu)

[Nandakumar-narayanan@uiowa.edu](mailto:Nandakumar-narayanan@uiowa.edu)

169 Newton Road

Department of Neurology

Roy J. and Lucille A. Carver College of Medicine

University of Iowa

Iowa City, IA 52242

**List of Supplementary Materials**

Supplementary Materials and Methods

Supplementary Results and Discussion related to Figure S2

Figure S1 to Figure S8

Table S1 to Table S11

**Supplementary MATERIALS AND METHODS**

**Yeast**

*Yeast culturing and TZ treatment :* WT and *snf1* mutant strains with a 3x integrated QUEEN reporter (MTY3264 and MTY3371 [1] were purchased from the Yeast Genetic Resource Centre Japan (YGRC). QUEEN reporter strains were grown to mid-log phase from fresh overnight cultures in synthetic complete (SC) media at 25 °C. The *snf1* mutant strain and the corresponding WT strain (BY4730) that were transformed with the Aβ42-GFP reporter (see plasmids below) were from the yeast deletion collection [2]. The transformed strains were grown to mid-log phase from fresh overnight cultures in SC-URA (SC media lacking uracil) media at 30 °C. Terazosin (TZ) HCl dihydrate was purchased from Selleck Biochemicals (S2059). Stocks were prepared at 20 mM in water and stored as aliquots at -80 °C. TZ was added to log phase cultures at final concentrations of 0–300 µM for dose response curves, with ATP being evaluated 60 minutes after each TZ addition. TZ was added at a final concentration of 40 µM to Aβ42-GFP reporter strains following 3 hours of reporter induction with CuSO_4_ (Fig. 1 and Fig. S1). Sixty minutes after TZ addition, GFP fluorescence, biochemical ATP values, and protein expression were evaluated as described below.

*Yeast culturing and Antimycin A treatment (Fig. S2)*: Wild type cells carrying the Aβ-GFP reporter or the QUEEN reporter were grown to early log phase and treated in parallel as follows. CuSO_4_ was added to 0.5 μM to induce Aβ42-GFP expression and cultures incubated for 2 hours at 30^o^C and 1 hour at 25^o^C. Cu^++^ was removed with two washes and the washed cultures resuspended and divided into two treatment groups: DMSO (0.1%) and DMSO + antimycin (50 μM in 0.1% DMSO). After 20 minutes, TZ was added to 30μM. Samples were imaged 20 minutes later and QUEEN ratios determined in one population and Aβ fluorescence in the other population after segmentation of the image.

*Yeast plasmids:* The Aβ42-GFP yeast expression plasmid was constructed by PCR amplification of the Aβ42-GFP cassette from plasmid aβ-GFP (pET28(A+)) (Addgene 72203) [3]. The PCR product was co-transformed into yeast along with a linearized yeast P_CUP1_ vector. The resulting recombinant construct, pGP2320, was isolated from yeast, confirmed by sequencing, and introduced into wild type and *snf1*Δ strains.

*Yeast microscopy and QUEEN ratiometric analysis:* Imaging was performed at 25 °C using fluorescence microscopy. Differential interference contrast (DIC), and two fluorescence images (Fluorescein (FITC) (ex480 image) and Ex409 (ex409 image)) were taken from multiple fields. The selection of fields was conducted under DIC to avoid unconscious bias. Open-source Fiji software was used to calculate the QUEEN ratio [4].

To measure GFP fluorescence of Aβ42-GFP, cells were circled in the DIC channel and regions of interest (ROIs) were measured in GFP for raw integrated density (“RawIntDen”) and area of the shape (“Area”). Similarly, five areas of the image background were measured to correct for background fluorescence in reported data points. The raw integrated density was divided by the area to establish fluorescence intensity for both cells and background in each field. The average background fluorescence intensity was subtracted from this value for each cell reported to provide the corrected fluorescence intensity (AU/mm^2^) reported in Fig 1D and Fig. S2. Fluorescence values are reported for cells having only soluble signal (i.e., no punctate GFP).

*Yeast biochemical ATP measurements:* Measurements of ATP in yeast cell extracts were performed as in [5] with minor modifications using the Invitrogen ATP Determination Kit (Invitrogen, Carlsbad, CA, #A22066). Briefly, extracts were prepared from late-log phase cultures (OD_600_ = 0.9–1.0), and the number of cells was quantified by hemocytometer. ATP was extracted by treating the pellets with a 10% trichloroacetic acid (TCA) solution. The cell suspension was vortexed for 1 minute and incubated on ice for 10 minutes. The pH of the suspension was adjusted to 7.2 by addition of 25 mM Tris/2 mM EDTA pH 9.4 to prevent inhibition of the luciferase reaction. Then 10 μl of extract was added to 90 μl of the Invitrogen kit assay mixture, and the ATP concentration was determined using an ATP standard curve. Luciferase was measured using a BioTek Cytation5 (Agilent, Santa Clara, CA) cell imaging multimode reader.

*Yeast extracts and western blot analysis:* Extracts from Aβ42-GFP-expressing yeast were prepared from log-phase cultures using a glass bead lysis method. Cultures used for imaging were then pelleted and frozen at -80 °C and used within 1 week. Approximately 100 µl of glass beads and 100 µl of extraction buffer (30 mM Tris, pH 8.5, 5 mM EDTA, 3 mM DTT, 5% glycerol plus 2.5 µl of 40 mM PMSF, and 2.5 µl of PIC; Sigma, P8215) were added to thawed pellets. Cells were disrupted with 3 × 60 seconds of Qiagen Tissue Lyser LT (Hilden, Germany) cycles alternating with 1 minute on ice. The mixture was centrifuged at 13,000 rpm for 1 minute, and the beads and pellet were discarded. The protein concentration was determined using the Bradford Protein Assay (Bio-Rad, Hercules, CA) with a bovine serum albumin (BSA) standard curve. Each extract was mixed with 5X LSB (Lauryl sulfate broth; 15% SDS, 0.575 M sucrose, 0.325 M Tris-HCl, pH 6.8, 5% β-mercaptoethanol, and 0.002% bromophenol blue) and incubated at room temperature (RT) for 15 minutes before being loaded on gradient Tris-Glycine polyacrylamide gels (Invitrogen) and subject to electrophoresis in Tris-glycine buffer + SDS at 100 volts for 90 minutes. Protein was transferred to nitrocellulose in Tris-glycine buffer (25 mM Tris, 192 mM glycine, 20% methanol) by electroblotting. Post-transfer membranes were incubated with Ponceau S for lane-to-lane normalization. Membranes were washed once in phosphate-buffered saline (PBS)-T (1X PBS with 0.1% Tween-20) to remove the stain prior to blocking. Membranes were blocked with 5% milk in 1X PBST for 1.5 hours at RT with rocking. α-GFP antibody (Proteintech, Rosemont, IL) was used at a 1:10,000 dilution. Secondary antibody (1:20,000) was IRDye 800CW goat anti-rabbit IgG (LI-COR). Membranes were imaged on the LI-COR Odyssey Fc imaging system in the 800 channels (IRDye800) with 2-minute exposure times. The GFP signal was normalized to the Ponceau S intensity on a per-lane basis.

**Mice**

*5xFAD mice:* All experimental procedures were performed in accordance with the relevant guidelines of Protocol #0062039 and with the approval of the Institutional Animal Care and Use Committee (IACUC) at the University of Iowa. 5xFAD transgenic mice (Tg(APPSwFlLon,PSEN1 *M146L*L286V)6799Vas) [6] and non-transgenic littermates were received from The Jackson Laboratory (Bar Harbor, ME) at approximately 5 weeks of age and were communally housed on a 12-hour light/dark cycle with *ad lib* access to laboratory rodent chow and water. To facilitate operant behavioral training (described below), mice were weighed daily and maintained on a restricted diet with *ad lib* access to water.

*TZ administration:* TZ (Tocris #1506, Minneapolis, MN) was delivered to mice through *ad lib* access to treated water in plastic water bottles, starting at 5 weeks of age and continuing until mice were euthanized. The vehicle control group had access to regular drinking water contained in the same type of plastic water bottles. Preparation of TZ began with a 41-mM stock solution (18.8 mg/ml of TZ HCl-dihydrate in water ~17.3 mg/ml of TZ HCl) stored at 4 °C for up to 1 week. We added 106 μL of stock solution, equating to 1.86 mg TZ HCl, to 300 ml water, to which mice had free access. The concentration of the final drinking water was 6.2 μg/ml TZ (1.9 mg/300 ml). An average mouse drinks ~5 ml of water per day [7], so each mouse consumed ~31 μg TZ daily (6.2 μg/ml x 5 ml/day). Additionally, experimental mice weigh on average 0.025 kg; thus, the TZ dose received by our animals was ~1.23 mg/kg/day (31μg/day/0.025 kg). All water bottles containing TZ or water were replaced every 2 days for the duration of the experiment until mice were euthanized [8].

*TZ dosing validation*

Five C57/bl6 mice were treated with water vs 5 C57/bl6 mice treated with TZ at ~1.23 mg/kg/day, for 3 weeks as described above. Water consumptions were monitored by weighing the water bottle daily. Mice were weighed daily. In this pilot study, the average rate of water consumption is 4.3ml/mouse/day (average weight 23.0 ± 1.2 g, mean ± SEM) in the water group and 4.4ml/mouse/day (average weight 22.0 ± 0.9 g) in the TZ group. The actual dose delivered is 1.27 mg/kg/day in the TZ group. Blood pressures were measured detailed as below. Mice were euthanized after 3 weeks of water vs TZ treatment, with plasma samples and flash frozen brain samples collected for TZ concentration measurement.

*Blood Pressure*

To minimize stress-related variability and improve blood pressure signal quality, animals were systematically acclimatized to the tail-cuff procedure. Beginning prior to the recording day, animals were placed in the restraining cylinders for short, repeated sessions each day. These acclimatization sessions familiarized the animals with the restraint and maintained tail circulation under warm conditions, allowing stable, reproducible blood pressure signals to be obtained. By the third day, animals tolerated the restraint without excessive movement, enabling reliable measurement cycles. Systolic blood pressure, diastolic blood pressure, and pulse rate were measured non-invasively on Day 3 using a tail-cuff photoplethysmographic system (BP-2000 Blood Pressure Analysis System, Visitech Systems, USA). During each measurement session, animals were placed in ventilated restraining cylinders positioned on a warming platform to promote tail blood flow. Following a minimum of five preliminary conditioning cycles, 10–20 measurement cycles were recorded for each animal. All readings were automatically captured. Cycles exhibiting motion artifacts, unstable waveforms, or irregular pulse detection were excluded according to standard tail-cuff quality criteria. Valid cycles were aggregated for each animal.

*Liquid chromatography-mass spectrometry (LC-MS) to measure mouse TZ plasma and brain concentration*

The TZ assay was performed at the Metabolomics Core at the University of Iowa as described previously[9]. Briefly, 90 µl of plasma samples were mixed with 1300 µl of the chilled extraction solvent consisting of methanol, acetonitrile, and water in a 2:2:1 ratio (volume/volume/volume) containing 3.9 ng/ml of deuterium-labeled TZ. 10 µl of 100% water was added to the mixtures. For brain samples, 50~80mg of flash frozen brain tissues were mixed with 800ul of the extraction solvent. Processed blank was prepared by adding only the extraction solvent to a microcentrifuge tube. Mixtures were vortexed for one minute and rotated at -20ºC for 1 hour to precipitate protein. Next, samples were centrifuged at 21,000 x g for 10 minutes at 4ᵒC to remove insoluble material. 1000 µl of supernatants (800ul in the case of brain samples) were transferred to clean microcentrifuge tubes and were evaporated to dryness using a Speed Vac and reconstituted in 25 µl of acetonitrile/water 1:1 ratio (v/v). Samples were stored at -20⁰C for 2 hours and then centrifuged at 21,000 x g for 10 minutes at 4ᵒC. The supernatant was transferred to LC autosampler vials for analysis. A standard dilution series was prepared in a commercially available human plasma sample (Innovative Research Inc, Novi, MI) for TZ as described under sample preparation and extraction where 10 μl of TZ dilution was added in place of water. For brain samples, we used flash frozen brain samples from untreated mice to prepare standard dilution series. Samples for the calibration curve were prepared in triplicate. Three sample were prepared without any spiked terazosin. We used mass spectrometry to quantify plasma TZ. LC-MS data were acquired on a Vanquish Flex UHPLC system (Thermo Scientific) coupled to a Thermo Q Exactive Hybrid Quadrupole-Orbitrap mass spectrometer. Two µL of metabolite extracts were separated using a Millipore SeQuant ZIC-pHILIC (2.1 X 150 mm, 5 µm particle size) column with a ZIC-pHILIC guard column (20 x 2.1 mm) attached to a Thermo Vanquish Flex UHPLC. Mobile phase was comprised of Solvent A: 20 mM ammonium carbonate, 0.1% ammonium hydroxide and Solvent B: acetonitrile. The mass spectrometer was operated in targeted Selected Ion Monitoring (tSIM), positive polarity mode from 0 to 10 minutes, with the spray voltage set to 3.0 kV, the heated capillary held at 275°C, and the HESI probe held at 350°C. The sheath gas flow was set to 40 units, the auxiliary gas flow was set to 15 units, and the sweep gas flow was set to 1 unit. MS data acquisition was performed in a range of m/z 70–1,000, with the resolution set at 70,000, the AGC target at 5 × 104, and the maximum injection time at 200 ms. The chromatographic gradient during tSIM acquisition time was at a flow rate of 0.15 mL/min during tSIM data acquisition at linear gradient from 80 to 20% Solvent B[10].

*Barnes maze spatial learning and memory assay:* At 8 months of age, mice were run on the Barnes maze assay in balanced cohorts using a 3-day optimized protocol for procedural learning and spatial memory acquisition [11]. Briefly, during the Barnes Maze assay, a mouse is placed on an elevated circular illuminated table with 10 ports arranged along the outer edge, one of which allows the mouse to escape to a preferred dark location—the target escape port. This protocol uses 3D-printed escape shuttles attached to the target escape port, combined with rotating the entire maze at each trial to reduce the possibility of unwanted cues interfering with spatial memory acquisition. On Day 1, mice performed one unanalyzed habituation trial and five spatial learning trials, with homecage bedding placed in the shuttle box at the bottom of all escape ports (target and non-target). The maze was turned every trial to minimize odor as a navigation cue. Intertrial intervals were 3 minutes from the time animals escaped. On Day 2, mice performed one probe trial to test early spatial retention ~24 hours after Day 1; on probe trials all ports were blocked to assess whether animals remember the location of the target escape port. Probe trials were followed by five spatial learning trials in which the escape port was unblocked. On Day 3, mice performed one probe trial to test spatial memory retention followed by five spatial learning trials. Data from the five spatial learning trials on each day were analyzed by averaging the distance travelled to reach the escape port. Data from the probe trials on Day 2 (“early”) and Day 3 (“late”) were analyzed as the percentage of time investigating the target spatial location, defined as a pie slice encompassing the target escape port and the two adjacent ports (3/10^th^ of the maze) during a 90-second probe, and compared to a theoretical mean of 30%.

*Switch interval timing assay:* Our mouse-optimized switch interval timing task is designed to capture an animal’s internal representation of time as detailed in our prior work [12–18]. Mice are trained to switch from a “short” to a “long” nosepoke after approximately 6 seconds. This switch response is an explicit time-based decision that requires working memory for temporal rules and attention to the passage of time and our group and others have shown that this version of interval timing models cognitive deficits associated with neurodegenerative disease in mice [15,19–21].

Briefly, mice were trained in standard operant chambers enclosed in sound-attenuated cabinets (MedAssociates, St. Albans, VT) that contained two light-equipped nosepoke response ports (left and right) on the front wall, a reward hopper (where food rewards are delivered) located between the two nosepoke response ports, and another light-equipped nosepoke on the back wall, opposite the reward hopper. Operant training began by “shaping” the animal’s behavior for 3 days. Trial initiation began with a response at the back nosepoke, at which point either the left or right front nosepoke was illuminated, and a response at the appropriate port resulted in a reward. After shaping, mice were then advanced to the interval timing switch protocol, for which each session was randomly organized into 50% short (6 seconds) and 50% long (18 seconds) trials. Either the left or right nosepoke was designated for short trials, and the contralateral nosepoke was designated for long trials (counterbalanced across mice and experimental groups). For both trial types, the trial is initiated with a back nosepoke response that generates two identical light cues above both the left and right nosepokes, along with an 8-kHz tone (72 dB). In short trials mice receive a reward after 6 seconds for the first response at the designated nosepoke. In long trials, mice begin by responding at the designated short-trial nosepoke but do not receive a reward. When there is no reinforcement after 6 seconds at the short-trial nosepoke, mice must switch to the designated long-trial nosepoke until a reward is delivered after 18 seconds. Median switch and CV times are plotted in Fig 2I-J. Only long switch trials were analyzed. Behavioral performance was analyzed for the last 4 days of training.

*Histology:* Mice were anesthetized with ketamine (100 mg/kg intraperitoneal (IP)) and xylazine (10 mg/kg IP) and transcardially perfused with cold PBS and 4% paraformaldehyde (PFA). Brains were removed and post-fixed in 4% PFA overnight, followed by immersion in a 30% sucrose in a PBS solution for at least 48 hours before 4–5-µm sections were collected and embedded in paraffin [15]. The Comparative Pathology Laboratory at the University of Iowa performed all sectioning and immunohistochemistry to visualize amyloid pathology in the mouse brain [22]. Antigen retrieval was done using citrate buffer (pH 6.0) in the decloaker (Biocare Medical, Pacheco, CA) at 110 °C for 15 minutes, followed by incubation in 6e10 monoclonal antibody (NBP2-62566, Novus, Centennial, CO) (1:5,000 in Dako diluent buffer) for 30 minutes at room temperature, and by incubation with the secondary antibody (anti-rabbit, HRP conjugated + Envision, Agilent) for 30 minutes. Sections were mounted, counterstained with hematoxylin for 3 minutes, and coverslipped. Slides were visualized using an Olympus BX53 microscope, DP73 digital camera, and CellSens Dimension Software (Olympus, Tokyo, Japan).

For amyloid pathology quantification, the pathologist (DM) examined tissues using the post-examination method of masking to group assignments [23]. To assess in an unbiased approach, the slides were masked and examined for immunostaining using specialized software (Olympus BX53 microscope, DP73 camera, and CellSens Dimension Software). Areas of interest were defined as hippocampus (CA1 region) and cortex (immediately adjacent to the CA1 region). These defined regions were evaluated with automated thresholding to detect 3,3'-Diaminobenzidine (DAB) staining, and the output was the percentage (%) of the area of tissue that was positive. As a quality control for each slide, the pathologist confirmed the automated assessment thresholds targeted the amyloid plaques (i.e. brown spots)[24]. The amyloid staining (% area) data were analyzed using the Graphpad Prism Software (Graphpad, San Deigo, CA).

*Mouse statistical analysis:* We analyzed effects of TZ in amyloid pathology and interval timing using linear mixed effects models (Weber et al, 2023). Evaluation of learning rate for Barnes was analyzed with pre-planned comparisons at each day. Probe trials were tested for statistical evidence of learning by the group, by comparing the percent time spent in the target quadrant to a theoretical mean (30%) representing chance performance (Bertolli et al., 2024). Four sessions of interval timing behavior were collected and analyzed per mouse. All data and statistical approaches were reviewed by the Biostatistics, Epidemiology, and Research Design Core (BERD) at the Institute for Clinical and Translational Science (ICTS) at the University of Iowa. All code and data are made available at: <http://narayanan.lab.uiowa.edu/article/datasets>. An α of p < 0.05 was considered significant.

*ADNI database analysis:* The Alzheimer’s Disease Neuroimaging Initiative (ADNI) is a multi-site observational study of patients with mild cognitive impairment (MCI) or AD. Data from the ADNI database are made publicly available, and at the time of our analysis, there were 2,343 participants. The ADNI study collects extensive data, including drug use, clinical outcome measures related to AD disease progression, and multiple neuroimaging sequences, among others.

In the current study, we aimed to compare clinical and neuroimaging outcome measures between eligible ADNI participants who were using TZ/DZ/AZ, tamsulosin, or none of these drugs. To be eligible for inclusion in these analyses, participants had to be male (females using TZ/DZ/AZ were less common and more likely to use these drugs for other indications); have more than one eligible ADNI visit; and carry a diagnosis of MCI or AD at their first eligible ADNI visit.

Use of drugs of interest (TZ/DZ/AZ or tamsulosin) was defined as at least 30 days of use during the time that the participant was enrolled in the ADNI dataset. If the participant started and stopped one of the drugs of interest prior to entering the study, they were not considered to be using the drug. Most participants were already taking the drug of interest at the time that they entered the study; however, some began treatment during their participation in the ADNI study. In these instances, the first visit that the participant was known to be taking the drug of interest was defined as the baseline visit. Concomitant use of TZ/DZ/AZ and tamsulosin was prohibited for these analyses. If a participant switched from one drug class to another, only the time of their first eligible exposure was included, and subsequent visits after switching were discarded.

Given that male participants who would be prescribed TZ/DZ/AZ or tamsulosin were of similar age and had similar comorbidities, all these participants were grouped together. We then performed 2:1 propensity score matching using a nearest neighbor technique to match users of TZ/DZ/AZ or tamsulosin to participants who had never been exposed to either drug group. Matching was based on baseline values of age, ADAS-Cog13 scores, CDR-SOB, baseline diagnosis (MCI or AD), and the number of copies of the APOE4 allele, which is a key modifier of AD recorded in the ADNI database.

Most participants were included in the ADNI study for 5 or fewer years. After matching, we constructed linear mixed effects regression models to compare the trajectory of change of the CDR-SOB, the ADAS-Cog13, and the mean annualized change in FDG-PET standardized uptake value from composite ROI of AD-relevant areas, among participants taking TZ/DZ/AZ or tamsulosin and matched participants who used none of these drugs. The models included a per participant intercept, and the predictor of interest was the group*duration interaction. Specifically, “duration” was the number of years in the ADNI study for each participant.

All analyses were performed in RStudio version 4.2.2. Matching procedures were carried out using the *MatchIt* package (version 4.5.0), and LMER analyses were performed using the *lmerTest* package (version 3.1.3). Results were considered significant if the *p* value was <0.05.

***Merative Marketscan administrative data of insurance claims***

*Cohort construction:* We used a new-user, active-comparator design. This is the gold standard design for observational pharmacoepidemiological investigations, as it reduces many important possible confounders, especially confounding by indication [25]. We identified all dispensing events for TZ, AZ, DZ, tamsulosin, finasteride and dutasteride. We grouped these drugs into three bins: TZ/DZ/AZ, tamsulosin, and finasteride/dutasteride. To ensure that we were identifying new users, we required at least 365 days of enrollment in the Merative Marketscan Commercial Claims and Encounters or Medicare Coordination of Benefits databases with prescription drug coverage before the first dispensing event. The first dispensing event was identified as our “index date,” and our “lookback period” was defined as the period before the index date, during which an individual was enrolled in an insurance plan contributing data to the Merative Marketscan database.

We then applied several exclusion criteria. First, the individual could not switch between the drug groups. Second, we required at least 1 day of follow-up after the index date. Third, these drugs are primarily used to treat BPH, with other indications being less common (TZ is used as a late-stage treatment for hypertension; tamsulosin can be used in the medical management of kidney stones). As a result, we decided to exclude female users. While most male individuals taking these drugs are likely using them for the management of BPH, no female users are managing BPH. Excluding women reduces unobserved heterogeneity, albeit at the cost of some representativeness of our sample. Fourth, because AD and BPH are mostly diseases of aging, we removed anyone who was younger than 40 years old on the index date. Finally, we removed anyone diagnosed with AD on or before the index date.

*Outcome definition:* We defined an individual as having AD if they ever had an ICD-9-CM diagnosis code of 046.11, 046.19, 290.10, 290.11, 290.12, 290.13, 290.20, 290.21, 290.3, 290.40, 290.41, 290.42, 290.43, 291.2, 294.10, 294.11, 331.0, 331.11, 331.19, 331.2, 331.82; or an ICD-10-CM diagnosis code of F01.50, F01.51, F03.90, F03.91, F04, G30.0, G30.1, G30.8, G30.9, G31.1, G31.09, G31.1, G31.81, G31.82, G31.83, G31.84, G31.85, G31.89, G31.9, I69.90, I69.910, I69.911, I69.913, I69.914, I69.915, I69.918, I69.919, I69.920, I69.921, I69.922, I69.923, I69.928, I69.931, I69.932, I69.933, I69.934, I69.939, I69.941, I69.942, I69.943, I69.962, I69.963, I69.964, I69.965, I69.969, I69.990, I69.991, I69.992, I69.993, I69.998, R41.0, R41.1, R41.2, R41.3, R41.4, R41.81, R41.82, R41.83, R41,840, R41.842, R41.843, R41.844, R41.89, R41.9. These codes were based on prior published validations of these code sets to identify AD. For our outcome, we used the first observed date in any setting—outpatient, inpatient, or facilities claim—with one or more of these diagnosis codes. The case-defining code set was harmonized with the prior published claims-based AD analyses[26], which have been validated against clinical adjudication. We acknowledge that administrative codes will inevitably apture a heterogeneous group that includes vascular dementia, mixed dementia, and other dementia syndromes alongside AD, and we now discuss this limitation explicitly. To address potential misclassification, we performed two sensitivity analyses: (i) restricting cases to specific AD ICD codes (G30.x and 331.0), and (ii) restricting to incident cases with at least two AD-coded encounters ≥ 30 days apart. The direction and approximate magnitude of the effect were preserved in both analyses, supporting robustness to outcome definition.

*Merative dataset statistical analysis*: We followed individuals from the first observed drug dispensing date (index date) until they were diagnosed with AD or left the Marketscan database, whichever happened first. If an individual left the Marketscan database for any reason, we censored their follow-up on the last observed enrollment date. To reduce confounding, we used inverse probability of treatment weighting (IPTW). IPTW weights each observation according to the inverse probability of the observed treatment outcome—that is, treated individuals have a weight of 1 / the probability of being treated, while untreated individuals have a weight of 1 / the probability of not being treated. After applying IPTW weights, a pseudo-population is derived, for which the values of the confounders used to calculate the treatment probability are more similar. Compared to propensity score matching, a related technique for addressing confounding, IPTW is less likely to create bias and is more data efficient, as it does not discard “unmatched” observations [27].

We used entropy balancing to calculate IPTW weights [28]. Entropy balancing calculates weights that minimize the negative entropy of the weights, resulting in the exact balance of the means of the included predictor variables. Unlike using a logistic regression model to estimate the weights, entropy balancing is more flexible and provides a closer-to-perfect balance. Additionally, entropy balancing can handle more than two classes, an important consideration given we have three groups (TZ/DZ/AZ, tamsulosin, and 5ARI). We used the following classes for entropy balancing: age; drug start year; average number of outpatient encounters per year during the lookback period; whether the patient had any hospitalizations during the lookback period; the number of inpatient encounters per year during the lookback period; the mean number of diagnoses per encounter date during the lookback period; whether the patient had PSA measured during the lookback period; whether the patient had a diagnosis of abnormal PSA; whether the patient had a diagnosis of BPH, slow urinary stream, orthostatic hypotension, or any other hypotension; whether the patient had a uroflow or cystometrogram collected during the lookback period; and the 29 AHRQ-Elixhauser comorbidity flags [29]. The large sample size yields extremely high power to detect clinically negligible differences and the resulting test statistics and p-values are largely uninformative (e.g., p < 10^-16^) regardless of the differences between the groups. We report the standardized mean difference (SMD), also known as Cohen’s d, as our measure of balance. The SMD is the difference between the groups on a particular variable divided by the standard deviation of that measure. Unlike the standard error, the standard deviation does not decrease simply by adding more observations. Common rules of thumb are that SMD with an absolute value of less than 0.1 are balanced, 0.2 are small differences, 0.4 are medium differences, and 0.6 are large differences.

We visualized differences in survival functions using the weighted Kaplan-Meier estimator. To quantify these differences, we estimated hazard ratios (HR) using weighted Cox proportional hazards regression. Because the standard errors of the Cox model do not account for the Kaplan-Meier weightings, we estimated standard errors, test statistics, p-values, and 95% CIs using fractional weighted bootstrapping with 999 bootstrap replicates. We assessed the validity of the proportional hazards using the correlation between the Schoenfeld residuals and time. Additionally, we estimated a model with time-varying coefficients at 1, 3, 5, 7.5, and 10 years. Kaplan-Meier weights were calculated using the R packages *ebal* and *WeightIt*; survival analysis was done using the R package *survival*; all analyses were done using R 4.1.

*Sensitivity analyses:*

We conducted several sensitivity analyses:

1) When calculating IPTW weights, extremely large weights can be calculated if the probability of the observed outcome is very small. This can result in a small number of observations having an oversized effect on the outcome. To combat this concern, we repeated our main analysis but trimmed our weights at the 2.5 and 97.5 percentiles, replacing more extreme observed treatment probabilities with the 2.5 and 97.5 percentiles.

2) We investigated whether excluding additional neurological diagnoses affected our results. There may be a period of time before the diagnosis of AD when there is clinical suspicion of AD. During this period, other tentative or more descriptive diagnoses, likely neurological in origin, may be applied to the patient. Specifically, we performed a sensitive analysis in which we excluded anyone with a diagnosis matching one of the Elixhauser neurological comorbidities during the lookback period. Additionally, we estimated an association among those with a neurological history. In both cases, the weights were re-estimated including the same factors as in the main model on the sub-cohorts with and without a neurological history. The HR was again estimated using Cox proportional hazards regression.

3) Prescribing may reflect health status evident to the prescriber but not readily captured in our data. Of specific concern is that a provider may elect to start a patient on tamsulosin or 5ARI, over TZ/DZ/AZ, based on some clinical factor that was unobserved in our data but that may be associated with increased risk of AD. Thus, we repeated our analysis, imposing a 1-year delay between the drug start date and the start of follow-up. This 1-year delay in the start of follow-up reduced potential confounding that may have arisen due to treatment selection factors being related to potential near-term AD risk.

4) Finally, we reviewed the role of additional diagnoses related to AD. We created our case definition to identify the most possible cases of AD. However, many of these AD cases may have been misdiagnosed or assigned as a placeholder diagnosis during a longer neurological course of care. Thus, we performed a sensitivity analysis in which cases of AD were required to include at least two diagnoses of AD, with at least 365 days between the first observed and last observed diagnosis date. We reasoned that when an AD diagnosis recurs, and more specifically, recurs over a long time span, it is more likely that a person had AD, as opposed to another non-AD disorder. If a person was diagnosed with AD but did not meet this heightened case definition, we censored their follow-up on the first observed diagnosis date.

*Negative control with brain malignant neoplasms:* Additionally, as a negative control where we do not expect TZ/DZ/AZ to have any protective effect, we considered malignant neoplasms of the brain (ICD-9-CM: 191.* and ICD-10-CM: C71.*). We did not distinguish between primary and metastatic tumors in the brain. As in the primary analysis, we estimated weights for treatment assignment. As the weighting is based on the treatment assignment and not outcome process, we used the same covariates in the weighting model as we do not expect treatment assignment to be different between these analytical cohorts. We then repeated the Cox proportional hazards modeling with 999 fractional weighted bootstrap replicates to estimate the standard error.

*E-values:* E-values for the hazard ratios were calculated using the R package EValue[30,31].

*Cox proportional hazards analysis:* We estimated a time-varying effect of use of TZ/DZ/AZ versus tamsulosin. For simplicity, 5ARI were not included in this analysis. We weighted the observations based on their IPTW estimated at the medication start date. The follow-up period was stratified based on days supplied of the medications into the groups [0, 180), [180, 365), [365, 548), [548, 720), [720, 1095) or [1095, infinity) days supplied. The coefficient associated with TZ/DZ/AZ was allowed to vary across those time periods. As before, we estimated 95% CI using fractional weighted bootstrapping.

**Supplementary RESULTS and DISCUSSION related to Figure S2.**

**TZ effects under an independent mitochondrial stress model (antimycin A)**

To extend our findings beyond the *snf1* mutant model of impaired energy regulation, we tested whether terazosin (TZ) protects against Aβ42-associated phenotypes under an independent mitochondrial stress condition. We used antimycin A, a well-characterized inhibitor of complex III of the electron transport chain, to induce acute mitochondrial dysfunction in wild-type yeast[4,32]. In this context, ATP was reduced and Aβ42-GFP fluorescence increased markedly relative to vehicle treated controls, suggesting a shift in proteostasis toward accumulation or concentration of fluorescent Aβ42 species.

Strikingly, addition of TZ following antimycin exposure significantly reduced Aβ42–sfGFP fluorescence toward baseline levels. Aβ42-sfGFP levels were not significantly affected by either antimycin A treatment or subsequent TZ exposure as determined by anti-GFP Western analysis following SDS-polyacrylamide gel electrophoresis. The effect of TZ on antimycin treated cells contrasts with the response observed in the *snf1*Δ model, in which TZ increased fluorescence by restoring folding capacity under chronic ATP limitation. These results indicate that TZ preserves proteostasis under both chronic metabolic stress (*snf1*Δ) and acute mitochondrial inhibition (antimycin A), but with distinct phenotypic outcomes depending on the underlying ATP-limiting condition.

The divergent responses observed across the two yeast models support a unified ATP-dependent model of Aβ42 proteostasis. In the *snf1*Δ background, chronic ATP depletion impairs protein folding, resulting in non-fluorescent, misfolded Aβ42 species that regain fluorescence upon ATP restoration by TZ. In contrast, under acute mitochondrial inhibition by antimycin, ATP depletion appears to trap pre-folded Aβ42-GFP in concentrated, fluorescent assemblies. TZ-mediated restoration of ATP reduces fluorescence in this setting, consistent with remodeling or dispersal of these assemblies rather than enhanced degradation. Thus, the apparent directionality of TZ effects on Aβ42 fluorescence reflects the underlying proteostatic state imposed by energy stress, rather than differences in aggregate burden per se. These findings suggest that TZ broadly stabilizes ATP-dependent proteostasis across mechanistically distinct energy-stress conditions.

**Supplementary Figures**

**Figure S1. (A)** Terazosin (TZ) does not raise ATP levels in a wild-type yeast strain. Each data point represents the QUEEN ratio of an individual cell (n=500 cells per dose). Significant changes in the QUEEN ratios at higher levels of TZ reflect a reduction, rather than an increase in ATP and may be indicative of mild toxicity. **(B)** Tamsulosin (TS) does not increase ATP levels. **(C)** Biochemical analysis of ATP showing that the CuSO_4_ used to induce Aβ42-GFP expression had no effect on ATP levels in wild-type or the *snf1* mutant; (n=6). Portions of this analysis are also shown in Fig. 1F. **(D)** Representative α-GFP Western blot analysis showing that the GFP levels are stable regardless of genotype and TZ treatment (n=4; p=0.98). Quantification was performed with reference to the Ponceau S total protein stain shown at the bottom. Significance for panels A-C was determined by ANOVA analysis with a post-hoc Tukey test (****, p<0.0001; ***, p< 0.001; **, p<0.01; *, p<0.05; ns, non-significant).

**Figure S2. Terazosin modulates Aβ42–GFP fluorescence under mitochondrial stress induced by antimycin A.** Wild-type yeast expressing Aβ42–GFP were treated with antimycin A to inhibit the electron transport chain and induce acute ATP depletion. Antimycin increased Aβ42–GFP fluorescence, while subsequent addition of TZ reduced fluorescence toward baseline. Quantification and analyses were performed across a minimum of 150 cells in 3-5 fields per treatment category in each of 3 independent experiments. Significance was assessed by one-way ANOVA with post-hoc Tukey test. These data demonstrate that TZ mitigates proteostasis defects under an independent mitochondrial stress condition distinct from the *snf1*Δ model. AU, arbitrary units; ns, non-significant; *, p≤0.01; ****, p≤0.0001.

**Figure S3.** A pilot study of TZ treatment in mice. Five wild type mice were treated with TZ (1.2mg/kg/day) vs 5 mice with water, for 3 weeks. A) TZ treatment did not significantly change blood pressure levels. B) We measured the plasma and brain TZ levels, and they are comparable to data from human subjects taking TZ at 5mg daily.

**Figure S4.** Amyloid (% area) pathology in the cortex region. A) example amyloid 6e10 staining images of a 5x FAD mouse (left) and a non-transgenic littermate (Right). B) TZ did not significantly reduce amyloid pathology in cortical regions of 5xFAD mice.

**Figure S5.** Total distance traveled in the Barnes maze during probe trials during early (Day 2) and late (Day 3) training. ~ = a main effect of distance with 5xFAD animals traveling less distance, with no effect of TZ or higher interactions for Early (F = 19.2; p = 0.001) and Late (F = 23.5, p = 0.00005) sessions.

**Figure S6.** **TZ target engagement.** The direct product of PGK1, 3-phosphoglycerate was significantly increased in FAD mice after TZ treatment (n=7-10 per group, p=0.024, Two-way ANOVA on log2(abundance) with Bonferroni correction).

**Figure S7.** Kaplan-Meier survival curves following medication initiation showing percent remaining free of any malignant neoplasm of the brain. Top panel shows the unadjusted survival curves while the bottom panel is estimated using IPTW weights.

**Figure S8.** **Latency to target in Barnes Maze, in the acquisition/learning phase and the probe phase.** Latency to target from the acquisition/learning phase and the probe phase plotted[33]. Latency was capped at 90 seconds.

**Table S1. Animal cohort characteristics (main cohort)**

| **Group** | **Start n** | **Attrition (cause)** | **End n** | **Body weight at 6 months of age (g, mean ± SD)** | **TZ dose (mg/kg/day)** |
| --- | --- | --- | --- | --- | --- |
| Water_NTg, F | 5 | 0 | 5 | 25.5 ± 2.5 | — |
| Water_NTg, M | 5 | 1=found dead in cage | 4 | 33.3 ± 6.0 | — |
| Water_FAD, F | 5 | 1=found dead in cage | 4 | 26.1 ± 3.4 | — |
| Water_FAD, M | 5 | 1=fight wound  1=found dead in cage | 3 | 33.7 ± 1.6 | — |
| TZ_NTg, F | 5 | 1=genotyping accident | 4 | 30.2 ± 4.8 | 1.2 |
| TZ_NTg, M | 5 | 0 | 5 | 37.9 ± 4.4 | 1.2 |
| TZ_FAD, F | 5 | 0 | 5 | 23.0 ± 4.8 | 1.2 |
| TZ_FAD, M | 5 | 0 | 5 | 37.4 ± 4.1 | 1.2 |
| **Water (pooled)** | **20** | **0** | **16** | **29.4 ± 5.3** | **—** |
| **TZ (pooled)** | 20 | **0** | **19** | **32.2 ± 7.4** | **1.2** |
| **p (TZ vs Water, pooled)** | **—** | **—** | **—** | **p = 0.19** | **—** |

**Table S2. Pilot cohort: terazosin pharmacokinetics and cardiovascular pharmacodynamics**

| **Group** | **Plasma TZ (ng/mL)** | **Brain TZ (ng/g tissue)** | **Tail-cuff SBP (mmHg)** | **Tail-cuff DBP (mmHg)** | **Water intake (mL/day)** | **Body weight**  **At beginning** | **TZ dose (mg/kg/day)** |
| --- | --- | --- | --- | --- | --- | --- | --- |
| Water (n = 5) | — | — | 116.6 ± 2.5 | 48.9 ± 1.8 | 4.3 | 23.0 ± 1.2 | — |
| TZ (n = 5) | 2.27 ± 0.33 (n=4) | 1.07 ± 0.09 | 115.1 ± 2.1 | 56.7 ± 3.7 | 4.4 | 22.0 ± 0.9 | 1.27 |

**Table S3. ADNI baseline demographics after matching**

|  | TZ/DZ/AZ | Tamsulosin | Neither | p-value |
| --- | --- | --- | --- | --- |
| N | 61 | 92 | 306 | N/A |
| Age, mean ± S.D. | 75.91 ± 6.53 | 76.32 ± 6.62 | 75.98 ± 6.49 | 0.895 |
| Baseline Dx, N (%)  MCI  Dementia | 48 (78.7)  13 (21.3) | 67 (72.8)  25 (27.2) | 237 (77.5)  69 (22.5) | 0.605 |
| APOE4 Alleles, N (%)  0  1  2 | 27 (44.3)  25 (41.0)  9 (14.8) | 44 (47.8)  38 (41.3)  10 (10.9) | 142 (46.7)  126 (40.8)  38 (12.4) | 0.969 |
| CDR-SB, mean ± S.D. | 2.18 ± 1.55 | 2.38 ± 2.10 | 2.22 ± 1.61 | 0.688 |
| ADAS-CoG 13,  mean ± S.D. | 20.36 ± 7.84 | 20.22 ± 9.72 | 19.90 ± 8.50 | 0.902 |
| MMSE, mean ± S.D. | 26.54 ± 2.55 | 26.27 ± 3.23 | 26.42 ± 2.67 | 0.830 |
| Digit Symbol Substitution, mean ± S.D. | 31.28 ± 8.97 | 34.77 ± 15.65 | 33.00 ± 11.62 | 0.488 |
| Hippocampal Volume (mL), mean ± S.D. | 6730 ± 1242 | 6499 ± 1197 | 6735 ± 1183 | 0.268 |

**Table S4. Numbers of ADNI participants by year.**

|  | **ADAS13** | | | **CDRSB** | | | **FDG** | | |
| --- | --- | --- | --- | --- | --- | --- | --- | --- | --- |
| **Year from Baseline** | **Control** | **Tamsulosin** | **TZ/DZ/AZ** | **Control** | **Tamsulosin** | **TZ/DZ/AZ** | **Control** | **Tamsulosin** | **TZ/DZ/AZ** |
| **0** | 308 | 93 | 61 | 308 | 93 | 61 | 244 | 53 | 44 |
| **1** | 276 | 70 | 49 | 280 | 70 | 49 | 66 | 17 | 16 |
| **2** | 199 | 42 | 31 | 207 | 47 | 32 | 103 | 21 | 13 |
| **3** | 136 | 31 | 20 | 141 | 34 | 21 | 29 | 4 | 5 |
| **4** | 81 | 13 | 14 | 81 | 15 | 14 | 11 | 0 | 3 |
| **5** | 14 | 3 | 3 | 15 | 4 | 3 | 6 | 1 | 1 |

**Table S5.** **Merative sample size by exclusion criteria**

|  | **TZ/DZ/AZ** | **Tamsulosin** | **5ARI** |
| --- | --- | --- | --- |
| **Users with 1+ Dispensing Events** | 1,028,561 | 2,854,989 | 653,508 |
| **Exclusive User** | 763,320 | 2,378,238 | 387,203 |
| **New User (At Least 365 Days of Lookback)** | 299,956 | 1,490,963 | 169,360 |
| **One or More Days of Follow-Up** | 299,764 | 1,489,812 | 169,212 |
| **Exclude Female Users** | 225,754 | 1,178,166 | 156,063 |
| **Exclude Age < 40** | 213,302 | 1,046,722 | 123,324 |
| **Exclude AD Case at Baseline** | 209,146 | 1,013,108 | 120,431 |

**Table S6. Merative baseline characteristics of new users at first observed claim for study drug**

|  | **Mean (SD)** | | | **Standardized Mean Differences - Unadjusted** | | |
| --- | --- | --- | --- | --- | --- | --- |
|  | **TZ/DZ/AZ** | **Tamsulosin** | **5ARI** | **TZ/DZ/AZ vs. Tamsulosin** | **TZ/DZ/AZ vs.**  **5ARI** | **Tamsulosin versus 5ARI** |
| **N** | 209,222 | 1,013,108 | 120,537 |  |  |  |
| **Lookback (Years)** | 3.96 (3.06) | 4.87 (3.65) | 4.11 (3.07) |  |  |  |
| **Follow-Up (Years)** | 2.84 (2.80) | 2.73 (2.66) | 2.93 (2.82) |  |  |  |
| **Age** | 61.96 (11.06) | 60.19 (11.16) | 61.46 (12.01) | 0.1560 | 0.0443 | 0.1117 |
| **Year** |  |  |  |  |  |  |
| **2002** | 5,608 (2.7%) | 12,623 (1.2%) | 1,423 (1.2%) | 0.0143 | 0.0150 | 0.0007 |
| **2003** | 5,335 (2.5%) | 15,889 (1.6%) | 2,216 (1.8%) | 0.0098 | 0.0071 | 0.0027 |
| **2004** | 15,915 (7.6%) | 17,377 (1.7%) | 3,019 (2.5%) | 0.0589 | 0.0510 | 0.0079 |
| **2005** | 16,477 (7.9%) | 26,515 (2.6%) | 5,106 (4.2%) | 0.0526 | 0.0364 | 0.0162 |
| **2006** | 11,048 (5.3%) | 30,965 (3.1%) | 5,853 (4.9%) | 0.0222 | 0.0042 | 0.0180 |
| **2007** | 12,980 (6.2%) | 37,256 (3.7%) | 7,479 (6.2%) | 0.0253 | 0.0000 | 0.0253 |
| **2008** | 16,605 (7.9%) | 43,943 (4.3%) | 9,951 (8.3%) | 0.0360 | 0.0032 | 0.0392 |
| **2009** | 20,216 (9.7%) | 58,552 (5.8%) | 13,589 (11.3%) | 0.0388 | 0.0161 | 0.0549 |
| **2010** | 17,659 (8.4%) | 63,388 (6.3%) | 12,054 (10.0%) | 0.0218 | 0.0156 | 0.0374 |
| **2011** | 14,033 (6.7%) | 75,491 (7.5%) | 10,990 (9.1%) | 0.0074 | 0.0241 | 0.0167 |
| **2012** | 14,178 (6.8%) | 87,533 (8.6%) | 9,557 (7.9%) | 0.0186 | 0.0115 | 0.0071 |
| **2013** | 11,884 (5.7%) | 77,878 (7.7%) | 7,484 (6.2%) | 0.0201 | 0.0053 | 0.0148 |
| **2014** | 9,565 (4.6%) | 77,843 (7.7%) | 6,527 (5.4%) | 0.0311 | 0.0084 | 0.0227 |
| **2015** | 7,740 (3.7%) | 69,478 (6.9%) | 5,418 (4.5%) | 0.0316 | 0.0080 | 0.0236 |
| **2016** | 6,306 (3.0%) | 65,490 (6.5%) | 4,413 (3.7%) | 0.0345 | 0.0065 | 0.0280 |
| **2017** | 5,152 (2.5%) | 54,700 (5.4%) | 3,531 (2.9%) | 0.0294 | 0.0047 | 0.0247 |
| **2018** | 4,728 (2.3%) | 49,424 (4.9%) | 3,187 (2.6%) | 0.0262 | 0.0038 | 0.0223 |
| **2019** | 5,078 (2.4%) | 53,240 (5.3%) | 3,098 (2.6%) | 0.0283 | 0.0014 | 0.0268 |
| **2020** | 4,145 (2.0%) | 46,044 (4.5%) | 2,545 (2.1%) | 0.0256 | 0.0013 | 0.0243 |
| **2021** | 4,570 (2.2%) | 49,479 (4.9%) | 3,097 (2.6%) | 0.0270 | 0.0039 | 0.0231 |
| **Outpatient Encounters Per Year** | 20.75 (25.70) | 22.50 (25.79) | 20.00 (22.93) | 0.0702 | 0.0304 | 0.1006 |
| **Ever Hospitalized** | 54,152 (25.9%) | 300,543 (29.7%) | 25,951 (21.5%) | 0.0378 | 0.0435 | 0.0814 |
| **Inpatient Encounters Per Year** | 1.13 (3.52) | 1.23 (3.67) | 0.79 (2.90) | 0.0293 | 0.1004 | 0.1296 |
| **Mean Number of Diagnoses Per Encounter Date** | 1.82 (0.84) | 2.15 (1.01) | 1.81 (0.77) | 0.3714 | 0.0180 | 0.3894 |
| **Elixhauser Comorbidities** |  |  |  |  |  |  |
| **Cardiac Arrhythmias** | 37,723 (18.0%) | 205,749 (20.3%) | 21,740 (18.0%) | 0.0228 | 0.0001 | 0.0227 |
| **Hypertension** | 144,069 (68.9%) | 594,329 (58.7%) | 63,780 (52.9%) | 0.1020 | 0.1595 | 0.0575 |
| **HTN with Complications** | 25,132 (12.0%) | 88,126 (8.7%) | 8,896 (7.4%) | 0.0331 | 0.0463 | 0.0132 |
| **Neurological Disease** | 8,867 (4.2%) | 50,473 (5.0%) | 4,808 (4.0%) | 0.0074 | 0.0025 | 0.0099 |
| **Diabetes** | 57,921 (27.7%) | 248,343 (24.6%) | 23.294 (19.3%) | 0.0307 | 0.0836 | 0.0529 |
| **Metastatic Cancer** | 2,884 (1.4%) | 22,544 (2.2%) | 1,683 (1.4%) | 0.0085 | 0.0002 | 0.0083 |
| **Solid Tumor** | 22,776 (10.9%) | 139,003 (13.7%) | 15,073 (12.5%) | 0.0283 | 0.0162 | 0.0122 |
| **COPD** | 41,925 (20.0%) | 224,732 (22.2%) | 21,875 (18.1%) | 0.0214 | 0.0189 | 0.0403 |
| **Depression** | 24,019 (11.5%) | 144,448 (14.3%) | 14,144 (11.7%) | 0.0278 | 0.0025 | 0.0252 |
| **Valvular Disease** | 23,263 (11.1%) | 120,527 (11.9%) | 13,611 (11.3%) | 0.0078 | 0.0017 | 0.0060 |
| **Renal Failure** | 21,543 (10.3%) | 65,964 (6.5%) | 5,954 (4.9%) | 0.0379 | 0.0536 | 0.0157 |
| **Psychoses** | 2,339 (1.1%) | 14,219 (1.4%) | 1,186 (1.0%) | 0.0029 | 0.0013 | 0.0042 |
| **Heart Failure** | 18,555 (8.9%) | 85,495 (8.4%) | 8,927 (7.4%) | 0.0043 | 0.0146 | 0.0103 |
| **Peripheral Vascular Disease** | 24,170 (11.6%) | 119,164 (11.8%) | 12,184 (10.1%) | 0.0021 | 0.0144 | 0.0165 |
| **Diabetes with Complications** | 23,763 (11.4%) | 101,787 (10.0%) | 7,858 (6.5%) | 0.0131 | 0.0484 | 0.0353 |
| **Rheumatoid Arthritis** | 9,280 (4.4%) | 55,231 (5.5%) | 5,044 (4.2%) | 0.0102 | 0.0025 | 0.0127 |
| **Obesity** | 23,646 (11.3%) | 146,625 (14.5%) | 8,742 (7.3%) | 0.0317 | 0.0405 | 0.0722 |
| **Fluid and Electrolyte Disorders** | 23,681 (11.3%) | 118,996 (11.7%) | 9,231 (7.7%) | 0.0043 | 0.0366 | 0.0409 |
| **Deficiency Anemia** | 11,700 (5.6%) | 58,554 (5.8%) | 5,870 (4.9%) | 0.0019 | 0.0072 | 0.0091 |
| **Hypothyroidism** | 18,910 (9.0%) | 105,013 (10.4%) | 11,820 (9.8%) | 0.0133 | 0.0077 | 0.0056 |
| **Liver Disease** | 14,741 (7.0%) | 104,508 (10.3%) | 7,754 (6.4%) | 0.0327 | 0.0061 | 0.0388 |
| **Peptic Ulcer Disease** | 3,316 (1.6%) | 18,652 (1.8%) | 1,632 (1.4%) | 0.0026 | 0.0023 | 0.0049 |
| **Blood Loss Anemia** | 2,545 (1.2%) | 13,846 (1.4%) | 1,335 (1.1%) | 0.0015 | 0.0011 | 0.0026 |
| **Weight Loss** | 6,159 (2.9%) | 41,391 (4.1%) | 3,638 (3.0%) | 0.0114 | 0.0007 | 0.0107 |
| **Pulmonary Circulatory Disorders** | 4,355 (2.1%) | 26,650 (2.6%) | 2,421 (2.0%) | 0.0055 | 0.0007 | 0.0062 |
| **Coagulopathy** | 6,396 (3.1%) | 39,889 (3.9%) | 3,658 (3.0%) | 0.0088 | 0.0002 | 0.0090 |
| **Alcohol Abuse** | 5,388 (2.6%) | 28,710 (2.8%) | 2,174 (1.8%) | 0.0026 | 0.0077 | 0.0103 |
| **HIV** | 887 (0.4%) | 4,012 (0.4%) | 852 (0.7%) | 0.0003 | 0.0028 | 0.0031 |
| **Paralysis** | 2,350 (1.1%) | 13,456 (1.3%) | 1,058 (0.9%) | 0.0020 | 0.0025 | 0.0045 |
| **Lymphoma** | 2,085 (1.0%) | 12,771 (1.3%) | 1,243 (1.0%) | 0.0026 | 0.0003 | 0.0023 |
| **Drug Abuse** | 3,460 (1.7%) | 19,260 (1.9%) | 1,242 (1.0%) | 0.0025 | 0.0062 | 0.0087 |
| **BPH Specific Diagnoses, Procedures** |  |  |  |  |  |  |
| **BPH Diagnosis** | 74,826 (35.8%) | 367,931 (36.3%) | 54,825 (45.5%) | 0.0055 | 0.0972 | 0.0917 |
| **Abnormal PSA Diagnosis** | 24,023 (11.5%) | 134,718 (13.3%) | 32,999 (27.4%) | 0.0182 | 0.1589 | 0.1408 |
| **PSA Measured** | 148,207 (70.8%) | 718,142 (71.0%) | 87,584 (72.7%) | 0.0015 | 0.0182 | 0.0168 |
| **Slow Stream Diagnosis** | 7,773 (2.8%) | 29,540 (2.9%) | 3,105 (2.6%) | 0.0016 | 0.0018 | 0.0034 |
| **Uroflow Procedure** | 21,715 (10.4%) | 105,615 (10.4%) | 12,827 (10.6%) | 0.0005 | 0.0026 | 0.0022 |
| **Cystometrogram Procedure** | 2,875 (1.4%) | 12,325 (1.2%) | 1,348 (1.1%) | 0.0016 | 0.0026 | 0.0010 |
| **Drug Specific Factors** |  |  |  |  |  |  |
| **Orthostatic Hypotension Diagnosis** | 1,678 (0.8%) | 9,797 (1.0%) | 1,150 (1.0%) | 0.0017 | 0.0015 | 0.0001 |
| **Other Hypotension Diagnosis** | 3,687 (1.8%) | 21,784 (2.2%) | 2,105 (1.7%) | 0.0039 | 0.0002 | 0.0040 |

**Table S7. Merative baseline characteristics of weighted pseudo-population**

|  | **TZ/DZ/AZ** | **Tamsulosin** | **5ARI** |
| --- | --- | --- | --- |
| **Effective Sample Size** | 143,949.2 | 982,281.2 | 82,725.3 |
| **Range of Weights (narrower is better)** | 0.0832 – 17.9876 | 0.3731 – 2.2825 | 0.0991 – 49.3096 |
| **Coefficient of Variability of Weights (smaller is better)** | 0.673 | 0.676 | 0.177 |
| **Lookback (Years)** | 4.47 (3.47) | 4.71 (3.54) | 4.41 (3.39) |
| **Follow-Up (Years)** | 2.55 (2.51) | 2.83 (2.77) | 2.68 (2.67) |
| **Age** | 60.57 (10.73) | 60.57 (11.23) | 60.57 (12.18) |
| **Year** |  |  |  |
| **2002** | 3,062.1 (1.5%) | 14,827.7 (1.5%) | 1,764.2 (1.5%) |
| **2003** | 3,652 (1.7%) | 17,684 (1.7%) | 2,104 (1.7%) |
| **2004** | 5,657.3 (2.7%) | 27,394.3 (2.7%) | 3,259.3 (2.7%) |
| **2005** | 7,493.8 (3.6%) | 36,111.9 (3.6%) | 4,296.5 (3.6%) |
| **2006** | 7,457.6 (3.6%) | 36,111.9 (3.6%) | 4,296.5 (3.6%) |
| **2007** | 8,992.1 (4.3%) | 43,542.3 (4.3%) | 5,180.6 (4.3%) |
| **2008** | 10,983.9 (5.2%) | 53,187 (5.2%) | 6,328.1 (5.2%) |
| **2009** | 14,389.4 (6.9%) | 69,677.5 (6.9%) | 8,290.1 (6.9%) |
| **2010** | 14,505.4 (6.9%) | 70,238.8 (6.9%) | 8,356.8 (6.9%) |
| **2011** | 15,660.3 (7.5%) | 75,831.4 (7.5%) | 9,022.2 (7.5%) |
| **2012** | 17,335.8 (8.3%) | 83,944.6 (8.3%) | 9,987.5 (8.3%) |
| **2013** | 15,151.2 (7.2%) | 73,365.9 (7.2%) | 8,728.9 (7.2%) |
| **2014** | 14,635.3 (7.0%) | 70,868 (7.0%) | 8,431.7 (7.0%) |
| **2015** | 12,874.9 (6.2%) | 62,343.6 (6.2%) | 7,417.5 (6.2%) |
| **2016** | 11,873.6 (5.7%) | 57,494.9 (5.7%) | 6,840.6 (5.7%) |
| **2017** | 9,875.2 (4.7%) | 47,818.5 (4.7%) | 6,689 (4.7%) |
| **2018** | 8,933.6 (4.3%) | 43,258.6 (4.3%) | 5,146.8 (4.3%) |
| **2019** | 9,568.8 (4.6%) | 46,334.5 (4.6%) | 5,512.8 (4.6%) |
| **2020** | 8,216.1 (3.9%) | 39,784.5 (3.9%) | 4,733.5 (3.9%) |
| **2021** | 8,903.5 (4.3%) | 43,113.0 (4.3%) | 5,129.5 (4.3%) |
| **Outpatient Encounters Per Year** | 22.00 (26.54) | 22.00 (25.41) | 22.00 (24.12) |
| **Ever Hospitalized** | 59,305.6 (28.3%) | 287,173.3 (28.3%) | 34,167.1 (28.3%) |
| **Inpatient Encounters Per Year** | 1.18 (3.55) | 1.18 (3.58) | 1.18 (4.31) |
| **Mean Number of Diagnoses Per Encounter Date** | 2.07 (1.09) | 2.07 (1.19) | 2.07 (0.94) |
| **Elixhauser Comorbidities** |  |  |  |
| **Cardiac Arrhythmias** | 41,320.7 (6.7%) | 200,085.6 (6.7%) | 23,805.7 (6.7%) |
| **Hypertension** | 124,913.2 (59.7%) | 605,192.5 (59.7%) | 71,909.3 (59.7%) |
| **Neurological Disease** | 9,990.5 (4.8%) | 48,403 (4.8%) | 5,751.3 (4.8%) |
| **Diabetes** | 51,472.5 (24.6%) | 249,379.2 (24.6%) | 29,631.4 (24.6%) |
| **Metastatic Cancer** | 4,221.6 (2.0%) | 20,453.2 (2.0%) | 2,430.3 (2.0%) |
| **Solid Tumor** | 27,538.5 (13.2%) | 133,421.3 (13.2%) | 15,853.2 (13.2%) |
| **COPD** | 44,954.0 (7.3%) | 217,679.1 (7.3%) | 25,898.9 (7.3%) |
| **Depression** | 28,451.2 (4.6%) | 137,768.4 (4.6%) | 16,391.3 (4.6%) |
| **Valvular Disease** | 24,505.7 (11.7%) | 118,727.9 (11.7%) | 14,107.3 (11.7%) |
| **HTN with Complications** | 19,020.3 (9.1%) | 92,151.3 (9.1%) | 10,949.5 (9.1%) |
| **Renal Failure** | 14,553.7 (7.0%) | 70,511.2 (7.0%) | 8,378.2 (7.0%) |
| **Psychoses** | 2,763.1 (1.3%) | 13,387.2 (1.3%) | 1,590.7 (1.3%) |
| **Heart Failure** | 17,590.9 (8.4%) | 85,226.4 (8.4%) | 10,126.6 (8.4%) |
| **Peripheral Vascular Disease** | 24,216.2 (11.6%) | 117,325.2 (11.6%) | 13,940.6 (11.6%) |
| **Diabetes with Complications** | 20,776.3 (9.9%) | 100,659.3 (9.9%) | 11,960.4 (9.9%) |
| **Rheumatoid Arthritis** | 10,831.3 (5.2%) | 52,476.5 (5.52%) | 6,235.3 (5.2%) |
| **Obesity** | 27,876.9 (13.3%) | 135,061.0 (13.3%) | 16,048 (13.3%) |
| **Fluid and Electrolyte Disorders** | 23,653 (11.3%) | 114,598.8 (11.3%) | 13,616.7 (11.3%) |
| **Deficiency Anemia** | 11,860.3 (1.9%) | 57,430.7 (1.9%) | 6,833 (1.9%) |
| **Hypothyroidism** | 21,135.8 (10.1%) | 102,400.9 (10.1%) | 12,167.3 (10.1%) |
| **Liver Disease** | 19,777.4 (9.5%) | 95,819.3 (9.5%) | 11,385.3 (9.5%) |
| **Peptic Ulcer Disease** | 3,674.7 (1.8%) | 17,803.8 (1.8%) | 2,115.5 (1.8%) |
| **Blood Loss Anemia** | 2,761.8 (0.4%) | 13,373.1 (0.4%) | 1,591.1 (0.4%) |
| **Weight Loss** | 7,971.1 (3.8%) | 38,619.2 (3.8%) | 4,588.7 (3.8%) |
| **Pulmonary Circulatory Disorders** | 5,203.7 (2.5%) | 25,211.6 (2.5%) | 2,995.7 (2.5%) |
| **Coagulopathy** | 7,781.2 (1.3%) | 37,678.8 (1.3%) | 25,898.9 (7.3%) |
| **Alcohol Abuse** | 5,651.3 (0.9%) | 27,364.9 (0.9%) | 3,255.8 (0.9%) |
| **HIV** | 895.7 (0.4%) | 4,339.6 (0.4%) | 515.6 (0.4%) |
| **Paralysis** | 2,625.2 (1.3%) | 12,718.6 (1.3%) | 1,511.2 (1.3%) |
| **Lymphoma** | 2,506.5 (1.2%) | 12,143.6 (1.2%) | 1,442.9 (1.2%) |
| **Drug Abuse** | 3,731.6 (1.8%) | 18,079.2 (1.8%) | 2,148.2 (1.8%) |
| **BPH Specific Diagnoses, Procedures** |  |  |  |
| **BPH Diagnosis** | 77,477.9 (37.1%) | 375,373.1 (37.1%) | 44,602 (37.1%) |
| **Abnormal PSA Diagnosis** | 29,853.1 (14.3%) | 144,635 (14.3%) | 17,185.6 (14.3%) |
| **PSA Measured** | 148,698.3 (71.1%) | 720,429.0 (71.1%) | 85,601.7 (71.1%) |
| **Slow Stream Diagnosis** | 5,941.7 (2.9%) | 28,980.8 (2.9%) | 3,443.5 (2.9%) |
| **Uroflow Procedure** | 21,824.7 (10.4%) | 105,738.4 (10.4%) | 12,563.9 (10.4%) |
| **Cystometrogram Procedure** | 2,575.9 (1.2%) | 12,480.2 (1.2%) | 1,482.9 (1.2%) |
| **Drug Specific Factors** |  |  |  |
| **Orthostatic Hypotension Diagnosis** | 1,966.3 (0.9%) | 9,526.7 (0.9%) | 1,132 (0.9%) |
| **Other Hypotension Diagnosis** | 4,294 (2.1%) | 20,804 (2.1%) | 2,471.9 (2.1%) |

**Table S8. Merative results of model with time-varying coefficients for TZ/DZ/AZ and 5ARI vs. tamsulosin**

|  | **Tam-sulosin** |  |  | **t** | **p-value** | **5ARI** |  |  | **t** | **p-value** |
| --- | --- | --- | --- | --- | --- | --- | --- | --- | --- | --- |
| **Years Since Index Date** | HR | LB | UB |  |  | HR | LB | UB |  |  |
| 0–1 | 0.860 | 0.808 | 0.916 | -4.7 | < 0.0001 | 0.979 | 0.908 | 1.056 | -0.5 | 0.5860 |
| 1–3 | 0.855 | 0.810 | 0.903 | -5.6 | < 0.0001 | 0.981 | 0.919 | 1.047 | -0.6 | 0.5553 |
| 3–5 | 0.796 | 0.843 | 0.852 | -6.5 | < 0.0001 | 0.917 | 0.845 | 0.994 | -2.1 | 0.0353 |
| 5–7.5 | 0.839 | 0.779 | 0.904 | -4.6 | < 0.0001 | 0.822 | 0.749 | 0.901 | -4.2 | < 0.0001 |
| 7.5–10 | 0.809 | 0.735 | 0.892 | -4.3 | < 0.0001 | 0.858 | 0.762 | 0.966 | -2.5 | 0.0114 |
| 10+ | 0.805 | 0.720 | 0.901 | -3.8 | < 0.0001 | 0.698 | 0.598 | 0.815 | -4.6 | < 0.0001 |

**Table S9. Merative Results of sensitivity analyses with re-defined cohorts, follow-up delays, and alternative case definitions**

|  | **Trimmed Weights** | **Positive Neurological History** | **No Neurological History** | **One Year Delay in Follow-Up** | **2+ Diagnoses, 365 days** |
| --- | --- | --- | --- | --- | --- |
| **Person-Years at Risk** |  |  |  |  |  |
| **TZ/DZ/AZ** | 512,670 | 19,411 | 514,504 | 521,884 | 533,753 |
| **Tamsulosin** | 2,870,250 | 118,478 | 2,751,358 | 2,683,610 | 2,870,393 |
| **5ARI** | 313,238 | 10,898 | 312,095 | 315,493 | 322,522 |
| **Cases of AD** |  |  |  |  |  |
| **TZ/DZ/AZ** | 6,665 | 1,208 | 5,875 | 5,197 | 1,760 |
| **Tamsulosin** | 47,225 | 8,204 | 39,266 | 34,262 | 13,452 |
| **5ARI** | 4,519 | 689 | 4,058 | 3,576 | 1,311 |
| **Incidence Rate** |  |  |  |  |  |
| **TZ/DZ/AZ** | 13.0 per 1,000 | 62.2 per 1,000 | 11.4 per 1,000 | 10.0 per 1,000 | 3.30 |
| **Tamsulosin** | 16.5 per 1,000 | 69.2 per 1,000 | 14.3 per 1,000 | 12.8 per 1,000 | 4.69 |
| **5ARI** | 14.4 per 1,000 | 63.2 per 1,000 | 13.0 per 1,000 | 11.3 per 1,000 | 4.06 |
| **HR (95% CI) [t, p-value]** |  |  |  |  |  |
| **TZ/DZ/AZ** | 0.806  (0.806, 0.806)  [-1,091.6, p < 10^-16^] | 0.897  (0.895, 0.900)  [-87.0, p < 10^-16^] | 0.825  (0.825, 0.826)  [-952.7, p < 10^-16^] | 0.823  (0.823, 0.824)  [-969.3, p < 10^-16^] | 0.726  (0.726, 0.727)  [-1,120.1, p < 10^-16^] |
| **Tamsulosin** | Reference | | | | |
| **5ARI** | 0.880  (0.880, 0.881)  [-653.3, p < 10^-16^] | 0.911  (0.909, 0.914)  [-64.7, p < 10^-16^] | 0.920  (0.920, 0.921)  [-445.2, p < 10^-16^] | 0.908  (0.907, 0.908)  [-492.1, p < 10^-16^] | 0.878  (0.877, 0.879)  [-293.4, p < 10^-16^] |

**Table S10. Merative E-values for unmeasured confounding and selection bias for the ever use analysis.** We report values both assuming and not assuming that AD is a rare disease. It is likely that for statistical purposes that AD is a rare disease (< 15% cumulative incidence at end-of-follow-up using the Kaplan-Meier estimator) but the HR = RR assumption would overstate E-values if the rare disease assumption does not hold. The E-values under both conditions are practically very similar.

| Comparison | E-Value Unmeasured Confounding | | E-Value Selection Bias | |
| --- | --- | --- | --- | --- |
|  | Rare Disease | Not Rare Disease | Rare Disease | Not Rare Disease |
| TZ/DZ/AZ versus Tamsulosin | 1.67 | 1.51 | 1.41 | 1.32 |
| 5ARI versus Tamsulosin | 1.36 | 1.28 | 1.23 | 1.19 |

**Table S11.** **Merative Cox proportional hazards analysis with time-varying coefficient of TZ/DZ/AZ use.**

| Days Supplied | Hazard ratio | 95% CI | |
| --- | --- | --- | --- |
|  |  | Lower Bound | Upper Bound |
| [0, 180) | 0.95 | 0.90 | 0.99 |
| [180, 365) | 0.72 | 0.67 | 0.77 |
| [365, 548) | 0.75 | 0.68 | 0.83 |
| [548, 720) | 0.59 | 0.52 | 0.68 |
| [720, 1095) | 0.65 | 0.58 | 0.72 |
| >= 1095 | 0.57 | 0.53 | 0.61 |

**References:**

1. Takaine M, Imamura H, Yoshida S. High and stable ATP levels prevent aberrant intracellular protein aggregation in yeast. Ron D, Holt LJ, editors. eLife. eLife Sciences Publications, Ltd; 2022;11:e67659. https://doi.org/10.7554/eLife.67659

2. Brachmann CB, Davies A, Cost GJ, Caputo E, Li J, Hieter P, et al. Designer deletion strains derived from Saccharomyces cerevisiae S288C: a useful set of strains and plasmids for PCR-mediated gene disruption and other applications. Yeast Chichester Engl. 1998;14:115–32. https://doi.org/10.1002/(SICI)1097-0061(19980130)14:2%3C115::AID-YEA204%3E3.0.CO;2-2

3. Wurth C, Guimard NK, Hecht MH. Mutations that Reduce Aggregation of the Alzheimer’s Aβ42 Peptide: an Unbiased Search for the Sequence Determinants of Aβ Amyloidogenesis. J Mol Biol. 2002;319:1279–90. https://doi.org/10.1016/S0022-2836(02)00399-6

4. Takaine M, Ueno M, Kitamura K, Imamura H, Yoshida S. Reliable imaging of ATP in living budding and fission yeast. J Cell Sci. 2019;132:jcs230649. https://doi.org/10.1242/jcs.230649

5. Xu L, Bretscher A. Rapid Glucose Depletion Immobilizes Active Myosin V on Stabilized Actin Cables. Curr Biol. Elsevier; 2014;24:2471–9. https://doi.org/10.1016/j.cub.2014.09.017

6. Oakley H, Cole SL, Logan S, Maus E, Shao P, Craft J, et al. Intraneuronal β-Amyloid Aggregates, Neurodegeneration, and Neuron Loss in Transgenic Mice with Five Familial Alzheimer’s Disease Mutations: Potential Factors in Amyloid Plaque Formation. J Neurosci. 2006;26:10129–40. https://doi.org/10.1523/JNEUROSCI.1202-06.2006

7. Nicolaus ML, Bergdall VK, Davis IC, Hickman-Davis JM. Effect of Ventilated Caging on Water Intake and Loss in 4 Strains of Laboratory Mice. J Am Assoc Lab Anim Sci JAALAS. 2016;55:525–33.

8. Cai R, Zhang Y, Simmering JE, Schultz JL, Li Y, Fernandez-Carasa I, et al. Enhancing glycolysis attenuates Parkinson’s disease progression in models and clinical databases. J Clin Invest. 2019;129:4539–49. https://doi.org/10.1172/JCI129987

9. Schultz JL, Gander PE, Workman CD, Boles Ponto LL, Cross S, Nance CS, et al. A dose-finding study shows terazosin enhanced energy metabolism in neurologically healthy adults. J Park Dis. 2025;15:1253–63. https://doi.org/10.1177/1877718X251356503

10. Cantor JR, Abu-Remaileh M, Kanarek N, Freinkman E, Gao X, Louissaint A, et al. Physiologic Medium Rewires Cellular Metabolism and Reveals Uric Acid as an Endogenous Inhibitor of UMP Synthase. Cell. 2017;169:258-272.e17. https://doi.org/10.1016/j.cell.2017.03.023

11. Bertolli A, Halhouli O, Liu-Martínez Y, Blaine B, Thangavel R, Zhang Q, et al. Renovating the Barnes maze for mouse models of Dementia with STARR FIELD: A 4-day protocol that probes learning rate, retention and cognitive flexibility [Internet]. bioRxiv; 2024 [cited 2024 Dec 10]. p. 2024.11.30.625516. https://doi.org/10.1101/2024.11.30.625516

12. Balci F, Papachristos EB, Gallistel CR, Brunner D, Gibson J, Shumyatsky GP. Interval timing in genetically modified mice: a simple paradigm. Genes Brain Behav. 2008;7:373–84. https://doi.org/10.1111/j.1601-183X.2007.00348.x

13. Bruce RA, Weber MA, Volkman RA, Oya M, Emmons EB, Kim Y, et al. Experience‐related enhancements in striatal temporal encoding. Eur J Neurosci. 2021;54:5063–74. https://doi.org/10.1111/ejn.15344

14. Bruce RA, Weber M, Bova A, Volkman R, Jacobs C, Sivakumar K, et al. Complementary cognitive roles for D2-MSNs and D1-MSNs during interval timing. Ding J, Wassum KM, editors. eLife. eLife Sciences Publications, Ltd; 2025;13:RP96287. https://doi.org/10.7554/eLife.96287

15. Larson T, Khandelwal V, Weber MA, Leidinger MR, Meyerholz DK, Narayanan NS, et al. Mice expressing P301S mutant human tau have deficits in interval timing. Behav Brain Res. 2022;432:113967. https://doi.org/10.1016/j.bbr.2022.113967

16. Tosun T, Gür E, Balcı F. Mice plan decision strategies based on previously learned time intervals, locations, and probabilities. Proc Natl Acad Sci. 2016;113:787–92. https://doi.org/10.1073/pnas.1518316113

17. Weber MA, Sivakumar K, Tabakovic EE, Oya M, Aldridge GM, Zhang Q, et al. Glycolysis-enhancing α1-adrenergic antagonists modify cognitive symptoms related to Parkinson’s disease. Npj Park Dis. Nature Publishing Group; 2023;9:1–7. https://doi.org/10.1038/s41531-023-00477-1

18. Weber MA, Kerr G, Thangavel R, Conlon MM, Gumusoglu SB, Gupta K, et al. Alpha-Synuclein Pre-Formed Fibrils Injected into Prefrontal Cortex Primarily Spread to Cortical and Subcortical Structures. J Park Dis. 2024;14:81–94. https://doi.org/10.3233/JPD-230129

19. Gür E, Duyan YA, Arkan S, Karson A, Balcı F. Interval timing deficits and their neurobiological correlates in aging mice. Neurobiol Aging. 2020;90:33–42. https://doi.org/10.1016/j.neurobiolaging.2020.02.021

20. Parker KL, Chen K-H, Kingyon JR, Cavanagh JF, Narayanan NS. Medial frontal ∼4-Hz activity in humans and rodents is attenuated in PD patients and in rodents with cortical dopamine depletion. J Neurophysiol. 2015;114:1310–20. https://doi.org/10.1152/jn.00412.2015

21. Singh A, Cole RC, Espinoza AI, Evans A, Cao S, Cavanagh JF, et al. Timing variability and midfrontal ~4 Hz rhythms correlate with cognition in Parkinson’s disease. Npj Park Dis. 2021;7:14. https://doi.org/10.1038/s41531-021-00158-x

22. Lee HY, Yoon S, Lee JH, Park K, Jung Y, Cho I, et al. Aryloxypropanolamine targets amyloid aggregates and reverses Alzheimer-like phenotypes in Alzheimer mouse models. Alzheimers Res Ther. 2022;14:177. https://doi.org/10.1186/s13195-022-01112-6

23. Meyerholz DK, Beck AP. Principles and approaches for reproducible scoring of tissue stains in research. Lab Invest. 2018;98:844–55. https://doi.org/10.1038/s41374-018-0057-0

24. Aeffner F, Bolon B, Boyle MH, Buetow BS, Forest T, Janardhan K, et al. Scientific and Regulatory Policy Committee Points to Consider*: Sample Selection, Assay Design, Data Generation and Interpretation, and Reporting Practices for Chromogenic Immunohistochemical (IHC) Assays in Nonclinical Drug Development. Toxicol Pathol. 2025;1926233251393154. https://doi.org/10.1177/01926233251393154

25. Lund JL, Richardson DB, Stürmer T. The Active Comparator, New User Study Design in Pharmacoepidemiology: Historical Foundations and Contemporary Application. Curr Epidemiol Rep. 2015;2:221–8. https://doi.org/10.1007/s40471-015-0053-5

26. Bhattacharyya J, Barnes LL, Chen Y, Gianattasio KZ, Grodstein F, James BD, et al. Evaluating linked ICD‐10 Medicare claims data as a method of dementia case ascertainment in research settings. Alzheimers Dement. 2025;21:e70200. https://doi.org/10.1002/alz.70200

27. Austin PC, Stuart EA. Moving towards best practice when using inverse probability of treatment weighting (IPTW) using the propensity score to estimate causal treatment effects in observational studies. Stat Med. 2015;34:3661–79. https://doi.org/10.1002/sim.6607

28. Hainmueller J. Entropy Balancing for Causal Effects: A Multivariate Reweighting Method to Produce Balanced Samples in Observational Studies. Polit Anal. 2012;20:25–46. https://doi.org/10.1093/pan/mpr025

29. van Walraven C, Austin PC, Jennings A, Quan H, Forster AJ. A Modification of the Elixhauser Comorbidity Measures Into a Point System for Hospital Death Using Administrative Data. Med Care. 2009;47:626. https://doi.org/10.1097/MLR.0b013e31819432e5

30. Smith LH, VanderWeele TJ. Bounding Bias Due to Selection. Epidemiology. 2019;30:509–16. https://doi.org/10.1097/EDE.0000000000001032

31. VanderWeele TJ, Ding P. Sensitivity Analysis in Observational Research: Introducing the E-Value. Ann Intern Med. 2017;167:268–74. https://doi.org/10.7326/M16-2607

32. Park WH. Antimycin A: From mitochondrial poison to multifaceted biological probe and therapeutic agent. Chem Biol Interact. 2025;422:111805. https://doi.org/10.1016/j.cbi.2025.111805

33. Bertolli A, Halhouli O, Liu-Martínez Y, Blaine B, Thangavel R, Zhang Q, et al. Renovating the Barnes maze for mouse models of Dementia with STARR FIELD: A 4-day protocol that probes learning rate, retention and cognitive flexibility [Internet]. 2024 [cited 2025 May 20]. https://doi.org/10.1101/2024.11.30.625516
